# Erucic acid utilization by *Lactobacillus johnsonii* N6.2

**DOI:** 10.3389/fmicb.2024.1476958

**Published:** 2024-11-25

**Authors:** Sharon C. Thompson, Reagan Beliakoff, Timothy J. Garrett, Claudio F. Gonzalez, Graciela L. Lorca

**Affiliations:** ^1^Department of Microbiology and Cell Science, Genetics Institute, Institute of Food and Agricultural Sciences, University of Florida, Gainesville, FL, United States; ^2^Department of Pathology, Immunology and Laboratory of Medicine, College of Medicine, University of Florida, Gainesville, FL, United States

**Keywords:** *Lactobacillus johnsonii*, erucic acid, long chain fatty acid, probiotic, nervonic acid

## Abstract

A multivariate nutritional analysis indicated that the consumption of erucic acid-rich food, a fatty acid (FA) found primarily in rapeseed and mustard oil, was positively correlated with higher counts of lactic acid bacteria (LAB). Furthermore, we showed *Lactobacillus johnsonii* N6.2, as well as other species of LAB tested from the former *Lactobacillus* genus, were able to efficiently use erucic acid (EA) as the source of FA. In this work, we identified significant changes induced in the FA profiles of *L. johnsonii* cultured with EA as the source of FA. We performed global transcriptomics to identify genes and pathways involved in EA utilization. It was found that *L. johnsonii* incorporates external fatty acids via a FakA/FakB and the *plsX/plsY/plsC* pathway for phosphatidic acid synthesis. It was found that cells grown in MRS with EA (MRS-E) significantly upregulated *fakB2* and *fakB4* when compared to cells grown in standard MRS with tween 80 as the source of FA. Additionally, in MRS-E, *L. johnsonii* N6.2 induced the expression of *plsY2, plsC2* and *plsC4* while the expression of *pslX* was constitutive during short term EA exposure. LC–MS analyses revealed that *L. johnsonii* N6.2 rapidly incorporates EA and synthesizes a variety of long chain fatty acids, including the health-relevant omega-9 monounsaturated fatty acids such as nervonic and gondoic acids.

## Introduction

Lactic acid bacteria (LAB) are known for their complex nutritional requirements. Depending on the genera, several nutrients and cofactors must be supplied to constitute a complex culture media to sustain their growth. Oleic acid (C18:1) was usually added to the media to supplement their auxotrophy for fatty acids (FA) (Hutchings and Boggiano, 1947). In 1960, the addition of Tween 80, mainly formulated with polysorbate-conjugated oleic acid (OA; C18:1), stimulated the growth of several species (Hutchings and Boggiano, 1947). Since then, lactobacilli have been routinely cultured in de Man, Rogosa-Sharpe (MRS) media that contains 0.1% Tween 80 as a source of FA. This culture media is required for all LAB species that lack a *de novo* fatty acid synthesis pathway (De Man et al., 1960). Several reports have analyzed the effects of supplementing MRS media with alternate FAs, such as linoleic or linolenic acids, on the FA profile of different *Lactobacilli* species (Kankaanpää et al., 2004). However, the FAs were added into MRS medium alongside Tween 80. Additionally, depending on the brand, commercial Tween 80 usually contains other fatty acids like linoleic, palmitic, and stearic acids as contaminants, making it difficult to extract conclusions (Zhang et al., 2012).

Understanding the pathway and biological mechanisms governing the assimilation, biosynthesis, and catabolism of FAs in LAB is relevant for human health as many species are used as probiotics. Microbial transformation of dietary FA by LAB plays a critical role in maintaining host gastrointestinal health, as was demonstrated in germ-free mice studies (Druart et al., 2015). We have recently reported that specific FAs present in the diet contribute to shaping the composition of the gastrointestinal microbiota (Thompson et al., 2023). We analyzed the dietary intake of participants in a double-blind, placebo-controlled, phase 1 clinical trial and found that specific dietary components correlated with high counts of LAB present in stool samples. A multivariate nutritional analysis indicated that the consumption of erucic acid-rich food, a FA found primarily in rapeseed and mustard oil, was positively correlated with higher counts of LAB (Thompson et al., 2023). Erucic acid (EA), a 22-carbon mono-unsaturated FA (MUFA) with a double-bound in the omega-9 position, is synthesized by many vegetables. Human consumption of EA has been restricted since 1970 based on scientific publications indicating that it produces cardiotoxicity in rats (Galanty et al., 2023). The formation of erucyl-carnitine inhibited the Krebs-cycle resulting in the accumulation of fatty acids and triglycerides in the heart of rodents. However, the potential toxicity in humans is still controversial and the limits allowed in different food products varies between countries.

We reported that specific species and strains of LAB, including *L. johnsonii* N6.2, have the capacity to utilize EA as the exogenous source of fatty acids (Thompson et al., 2023). *L. johnsonii* N6.2 was selected for further analyses due to its significant probiotic capabilities previously reported in animal models and human subjects. In rodents, the administration of *L. johnsonii* N6.2 mitigated the onset of type 1 diabetes while in healthy subjects, the probiotic administration was found to improve gut health while modulating immune responses (Valladares et al., 2010; Marcial et al., 2017). We thus hypothesize that the utilization and eventual transformation of EA by *L. johnsonii* N6.2 may help to lower its availability in food components, transforming it into health relevant bioactive compounds thereby contributing to its probiotic capabilities in improving the host’s health.

Hence, the data obtained from this clinical trial triggered the study of EA metabolism in *L. johnsonii* as the sole FA in a modified MRS media. We reported that *L. johnsonii* N6.2 as well as all other species of LAB tested from the former *Lactobacillus* genus were able to efficiently use EA as the source of FA (Thompson et al., 2023). However, the use of alternate exogenous FA such as EA will induce changes in the bacterium physiology, affecting the overall FA profile. These changes may improve or hinder some of the probiotic characteristics previously reported. For example, alterations in the membrane lipid composition such as reduced saturated-to-unsaturated fatty acid ratios have been associated with modification in microbial adhesion and stress tolerance in *Lactobacillaceae* (Kankaanpää et al., 2004; Muller et al., 2011). Our laboratory has recently shown that lipids from *L. johnsonii* N6.2 are effectors of its probiotic abilities (Cuaycal et al., 2023). This strain was able to mitigate the onset of Type One Diabetes (T1D) in animal models and to boost immunity (Valladares et al., 2010; Teixeira et al., 2018; Teixeira et al., 2021). One of the key mechanisms promoting health could be related to an overall decrease in systemic inflammation (Valladares et al., 2010; Marcial et al., 2017). In this context, we recently showed that FAs and lipids from *L. johnsonii* N6.2 play a critical role by modulating dendritic cells immune response (Cuaycal et al., 2023).

In this work, we assessed the FAs produced by *L. johnsonii* when cultivated with a singular exogenous FA, EA. We expect that the information gained will help guide future immunological studies. Here, we evaluated the changes induced in the FA profiles of cells cultured with EA as the source of FA and the profiles obtained were compared to those from cells grown on Tween 80 and oleic acid (OA). We performed global transcriptomics to identify genes and pathways involved in EA utilization. The identified genes were associated to a metabolic pathway to explain the variations of the FA profiles determined in the cells. It was found that *L. johnsonii* incorporates external fatty acids via a FakA/FakB mechanism. Interestingly, *L. johnsonii* N6.2 synthesizes a variety of long chain fatty acids, including the health-relevant omega-9 monounsaturated fatty acids like nervonic and gondoic acids. Based on gene expression, EA is expected to be modified to generate complex phospholipids with varying chain lengths and saturation degrees.

## Materials and methods

### Bacterial growth

*L. johnsonii* N6.2 was routinely grown in standard MRS with tween 80 (MRS-T) containing: 10 g/L peptone, 10 g/L beef extract (Bacto), 5 g/L yeast extract (Bacto), 20 g/L glucose, 2 g/L di-potassium hydrogen phosphate, 5 g/L sodium acetate, 2 g/L ammonium citrate tribasic, 0.2 g/L magnesium sulfate, 0.05 g/L manganese sulfate, 1 mL/L (Tween 80) for 16 h at 37°C. Alternate formulations of MRS without Tween 80 (MRS-NT) were supplemented with 0.1% oleic acid (C18:1) (MRS-O) or 0.1% EA (C22:1) (MRS-E) as described previously using 1% DMSO as a vehicle (Thompson et al., 2023). As a vehicle control, MRS-T with 1% DMSO (MRS-TD) was used. Tween 80 (catalog #BP338-500) and oleic acid (catalog #A195-500) were obtained from Fisher Bioreagents while EA (catalog #D0965) was obtained from TCI America.

For fatty acid analysis, *L. johnsonii* N6.2 were grown twice in MRS-T for 16 h at 37°C. Cells were pelleted and washed with MRS-NT to avoid carryover of residual Tween80. Next, cultures were inoculated at OD_600_ = 0.05 in either MRS-E, MRS-O, or MRS-TD. Cultures were incubated at 37°C for approximately 6–8 h to reach an OD600 = 0.5 and immediately collected by centrifugation at 3000 x rpm for 15 min. For fatty acid analyses, the pellets were washed twice with 0.1% peptone water and twice with PBS. When indicated, cells grown in MRS-E and MRS-O were likewise collected by centrifugation, washed twice with 0.1% peptone water, and then resuspended in the opposite media (MRS-E in MRS-O, and vice versa). Cells were incubated at 37°C for 10 min, collected by centrifugation, and washed twice with PBS. All cells were then stored at −80°C until further analysis. For fatty acid analysis, cells were lyophilized using the Labconco FreeZone for 24 h and stored at −80°C until further analysis.

For timepoint analysis of gene expression, cells were grown in MRS-TD to mid-log phase (OD_600_ = 0.6) and collected by centrifugation. Cells were then washed twice with 0.1% peptone and resuspended in MRS-TD, MRS-O, or MRS-E media and incubated for 5, 15, 30, and 60 min at 37°C. At the end of each timepoint, cells were immediately centrifuged at 13,000 rpm for 1 min, flash frozen and stored at −80°C for further analysis.

### Total fatty acid analysis

*L. johnsonii* N6.2 cells were collected, lyophilized, and stored as described above for fatty acid extraction and analysis performed by University of Florida Southeast Center for Integrated Metabolomics (SECIM). All reagents used were of LC–MS grade and obtained from Fisher Scientific. For total fatty acid analysis, fatty acid extractions were performed using 100 mg samples of lyophilized cell pellets weighed in a clean, conical glass tube (5 mL volume). To the lyophilized cell pellets, 1 mL acetonitrile containing 100 mg/L butylated hydroxy toluene (BHT) was added, and the sample was sonicated for 5 min to mix. Next, 2 mL hexane was added, and the sample was centrifuged at 3,260 x g for 1 min. The top layer (1.5 mL) was removed and transferred to a clean glass culture tube and was dried under a gentle stream of nitrogen. The dried sample was reconstituted in 0.5 mL 80/20 acetonitrile/5 mM ammonium acetate, and 10 μL of the injection standard mixture (Docosahexaenoic acid-D5, Eicosapentaenoic Acid-D5, α-Linolenic Acid-D14) was added. The final sample was transferred to an LC vial for LC–MS analysis.

Fatty acid analysis was performed on a Thermo Q-Exactive Orbitrap Mass Spectrometry with Dionex Ultimate 3,000 UHPLC and autosampler. Separation was achieved on a Waters HSS T3 column (150 × 2.1 mm, 1.8 μm) at a flow rate of 0.5 mL/min and column temperature of 30°*C. mobile* phase A and B consisted of 1 mM ammonium acetate in water and 0.1% acetic acid in acetonitrile, respectively. Gradient elution started at 25% A and 75% B from 0 to 0.5 min with a linear increase to 90% B from 0.5 to 7 min, and a further increase to 95% B from 7 to 8 min then held at a constant 95% B from 8 to 21 min. The column was then returned to initial conditions in 0.5 min and equilibrated for 4.5 min. The mass spectrometry was operated in negative heated electrospray ionization (H-ESI) mode with a resolution setting of 70,000 at m/z 200, collecting m/z 100–700. Data-dependent analysis was conducted on the top 5 most abundant peaks throughout the run with a resolution setting of 17,500, ion time of 75 ms, and collision-induced dissociation of 30, 50, and 70. The HESI settings were 3 kV, 100°C probe temperature, 300°C capillary temperature, 40 arb sheath gas, 5 arb auxiliary gas, and 1 arb sweep gas. Fatty acids were identified by accurate mass (<10 ppm accuracy), retention time, and tandem mass spectrometry.

### DNA and RNA extraction

*L. johnsonii* N6.2 were grown and collected as described, and total DNA was extracted using the DNeasy Blood and Tissue kit (Qiagen) utilizing the manufacturer’s protocol for gram-positive bacteria. For RNA extraction, cells were harvested and frozen at −80°C (Beliakoff et al., 2024). Briefly, 100 mg *L. johnsonii* N6.2 pellets were resuspended in Trizol and the suspension was added to 100 mg ice-cold zirconia beads. Cell lysis was performed by vortexing each tube for 5 30-s increments, resting the tubes on ice in between each round of vortexing. To remove the beads, the mixtures were centrifuged at 12,000 x g for 10 min at 4°C, and the supernatant was transferred to a fresh tube and incubated at room-temperature for 5 min. Next, 0.2 X volume of chloroform was added followed by manual shaking for 15 s, and then a 10-min incubation at room temperature. The samples were centrifuged at 12,000 x g for 15 min at 4°C, and the top aqueous phase was transferred to a fresh tube. Then, 1 X volume of 100% ethanol was added, and the mixture was loaded into spin columns from the RiboPure-Bacteria kit (Invitrogen, Waltham, MA). Subsequent wash steps were performed following the manufacturer’s instructions. DNA was removed from RNA extractions via the Invitrogen TURBO DNase kit (Thermo Fisher Scientific) following manufacturer’s instructions. The integrity of RNA extractions was determined utilizing 1% agarose gels, and RNA was quantified using Thermo Scientific Nanodrop One Microvolume UV–vis spectrophotometer (Thermo Fisher Scientific).

### RNA-Seq

For RNA-Seq analysis, cells were harvested at mid-exponential phase, around 6 h of growth, to an OD_600_ = 0.7 for MRS-TD and OD_600_ = 0.5 for MRS-E. RNA-Seq library preparation and sequencing was performed by Novogene (Novogene Co., Davis, CA, USA) as previously described (Beliakoff et al., 2024; da Silva et al., 2023). Following generation of clusters, library preparations were sequenced on an Illumina platform and paired-end reads were generated. Briefly, raw reads of FASTQ were processed through fastp (Chen et al., 2018). Reference genome (NC_022909.130) and gene model annotation files from NCBI were downloaded, and Bowtie2 was utilized to construct the reference genome index and align the clean reads (Langmead and Salzberg, 2012). To count the read numbers mapped to each gene, the program FeatureCounts was used (Liao et al., 2014). DESeq2 was used to analyze and determine differential expression with resulting *p*-values adjusted via the Benjamini and Hockberg’s approach for false discovery rate (Benjamini and Hochberg, 1995; Anders and Huber, 2010). Differentially expressed genes were assigned as those found by DESeq2 to have an adjusted *p* < 0.05. To identify Gene Ontology (GO) enrichment of differentially expressed genes, the clusterProfiler R pachage was used and gene length bias was corrected (Wu et al., 2021). Significant enrichment of GO terms by differentially expressed genes were defined by *p* < 0.05, and clusterProfiler was used to test the statistical enrichment of differentially expressed genes in the Kyoto Encyclopedia of Genes and Genomes (KEGG) pathways (Kanehisa et al., 2008).

### Quantitative real time PCR analysis

Quantitative real time PCR (qRT-PCR) analysis was performed following RNA extraction as described above. cDNA synthesis was performed using the iScript cDNA Synthesis kit (Bio-Rad) following manufacturer protocols. qRT-PCR was performed using PowerUp SYBR Green Master Mix (Applied Biosystems) in a QuantStudio 6 machine (Applied Biosystems). Changes in gene expression (CT values) were determined for each condition described above (MRS-O 5, 15, 30, and 60 min; MRS-E 5, 15, 30, and 60 min; and MRS-TD 5, 15, 30, and 60 min). The CT values of MRS-TD 5 min were utilized as the control by which all other conditions were compared using the 2^-ΔΔCT^ method. For the internal control, expression of the *rpoD* gene was used. All primers used are described in Supplementary Table S1.

## Results

### *L. johnsonii* N6.2 fatty acids pool is significantly changed by growth in erucic acid

Previously, *L. johnsonii* N6.2 was found to utilize the alternate fatty acid EA as the FA source. However, significant differences in growth kinetics were observed when compared to growth in OA or standard media that contained tween 80 (Thompson et al., 2023). It was thus hypothesized that *L. johnsonii* N6.2 may alter both its gene expression and fatty acid composition when utilizing this alternate fatty acid. To evaluate impact of EA on FA composition, *L. johnsonii* was grown in MRS-E and MRS-O. Growth in presence of Tween 80 with 1% DMSO as the fatty acid vehicle control (MRS-TD) was used as a reference. In parallel, the presence of contaminants and traces in the pure EA, OA, and tween 80 used to amend the media was determined. The FA profile of *L. johnsonii* N6.2 grown in standard conditions (MRS-TD) was significantly different compared to MRS-E and MRS-O (Table 1). The composition of the FA extracted from cells grown in MRS-TD were: oleic acid (18:1) 40.5%, linoleic acid (18:2) 12.1%, palmitic acid (16:0) 10.4%, and phytomonic acid (also known as lactobacillic acid) (19:0cyc11) 22%. *L. johnsonii* N6.2 grown in MRS-E was rich in EA (22:1) 25%, OA (18:1), 18.3%, docosadienoic acid (22:2) 7.9%, and phytomonic acid (19:0cyc11) 6.1%. In MRS-O, *L. johnsonii* N6.2 fatty acids consisted of oleic acid (18:1) 16.9%, palmitoleic acid (16:1) 10.7%, linoleic acid (18:2) 23.5%, myristic acid (14:0) 12.7%, palmitic acid (16:0) 13.4%, and phytomonic acid (19,0cyc11) 9.6%. The comparative analyses of the cells grown in the FA was compared to the source FA used to supplement the media. We found that the fatty acids detected in cells growing in MRS-TD and MRS-O could be assimilated from the media since the analysis of OA and Tween 80 used in our assays indicated that all of them were present in the OA or Tween 80 added to the media. However, in the case of MRS-E, only docosadienoic acid was detected in traces on the commercial EA used to supplement the MRS (Table 1). These results indicate that a distinct fatty acid pool is enriched when *L. johnsonii* N6.2 is grown in EA.

**Table 1:** Fatty acid profile of *L. johnsonii* N6.2 cells grown in MRS-TD, MRS-E, and MRS-O.

Fatty acids	CT	MRS-TD	CE	MRS-E	CO	MRS-O
Saturated						
Caprylic acid (8:0)	ND	0.2 ± 0.03 ^a^	ND	0.4 ± 0.03^a^	ND	0.05 ± 0.03^b^
Pelargonic acid (9:0)	ND	0.03 ± 0.01 ^a^	ND	0.02 ± 0.01^b^	ND	0.01 ± 0^b^
Undecylic acid (11:0)	ND	0.01 ± 0^a^	ND	0.1 ± 0^b^	ND	ND^a^
Lauric acid (12:0)	ND	0.7 ± 0.1^a^	ND	1.4 ± 0.1^b^	0.03	0.2 ± 0.03^a^
Tridecylic acid (13:0)	ND	0.02 ± 0^a^	ND	0.1 ± 0.02^a^	0.01	0.1 ± 0.01^a^
Myristic acid (14:0)	0.1	1.3 ± 0.3^a^	ND	0.6 ± 0.1^a^	2.3	12.7 ± 1.3^b^
Pentadecylic acid (15:0)	0.1	0.2 ± 0.03^a^	ND	0.1 ± 0.01^a^	0.3	2.4 ± 0.5^b^
Palmitic acid (16:0)	0.1	10.4 ± 0.6^a^	ND	7.6 ± 0.3^b^	0.8	13.4 ± 1.6^a^
Margaric acid (17:0)	3.1	1.2 ± 0.1^a^	ND	0.2 ± 0.0^b^	5.7	0.6 ± 0.05^b^
Stearic acid (18:0)	7.2	6.8 ± 0.5^a^	14.3	2.1 ± 0.2^b^	1.4	1.6 ± 0.2^b^
Nonadecylic acid (19:0)	0.1	0.1 ± 0.01^a^	ND	0.01 ± 0^b^	0.01	0.03 ± 0^b^
Arachidic acid (20:0)	0.1	0.02 ± 0^a^	ND	1.1 ± 0.1^b^	0.02	ND^a^
Behenic acid (22:0)	ND	0.01 ± 0^a^	2.0	1.7 ± 0.4^ab^	ND	ND^b^
Phytomonic acid (19:0cyc11)	0.1	22.0 ± 0.6^a^	ND	6.1 ± 0.3^b^	1.2	9.6 ± 1.1^c^
Monounsaturated						
Palmitoleic acid (16:1)	0.06	3.4 ± 0.6^ab^	ND	1.7 ± 0.1^a^	0.8	10.7 ± 0.3^b^
Oleic acid (18:1)	86.8	40.5 ± 0.9^a^	ND	18.3 ± 0.06 ^b^	48.4	16.9 ± 3.3^b^
Erucic acid (22:1)	0.4	0.2 ± 0.09^a^	82.21	25.0 ± 0.9^b^	0.01	0.2 ± 0.09^a^
Nervonic acid (24:1)	ND	0.0^a^	ND	3.5 ± 0.7^b^	ND	ND^a^
Gadoleic acid (20:1 n-11)	ND	0.03 ± 0.05^a^	ND	0.7 ± 0.06 ^ab^	0.1	0.4 ± 0.02^b^
Gondoic acid (20:1 n-9)	ND	0.2 ± 0.01^a^	ND	6.8 ± 0.6 ^ab^	1.8	2.2 ± 0.07^b^
Polyunsaturated						
Linoleic acid (18:2)	ND	12.1 ± 0.5^a^	ND	6.0 ± 0.5^a^	22.6	23.5 ± 1.4^a^
Stearidonic acid (18:4)	ND	0.01 ± 0^a^	ND	0.2 ± 0.03^b^	0.01	0.05 ± 0.02^a^
Dihomo-linoleic acid (20:2)	ND	0.05 ± 0.01^a^	ND	2.2 ± 0.1^a^	0.8	1.3 ± 0.05^a^
Eicosatrienoic acid (20:3)	ND	0.2 ± 0.02^ab^	ND	0.5 ± 0.03^a^	0.7	1.6 ± 0.1b
Eicosatetraenoic acid (20:4)	ND	0.2 ± 0.1^a^	ND	1.3 ± 0.1^b^	0.3	0.6 ± 0.03^a^
Timnodonic acid (20:5)	ND	0.01 ± 0^a^	ND	0.08 ± 0^b^	0.01	0.02 ± 0^a^
Docosadienoic acid (22:2)	ND	0.01 ± 0^a^	1.5	7.9 ± 0.2^b^	ND	0.02 ± 0^a^
Docosatrienoic acid (22:3)	ND	ND^a^	ND	4.0 ± 0.2^b^	ND	ND^a^
Ardenic acid (22:4)	ND	ND^a^	ND	0.05 ± 0^b^	0.01	ND^a^
Docosahexaenoic acid (22:6)	ND	ND^a^	ND	0.01 ± 0^b^	ND	ND^a^
Docosapentaenoic acid (22:5w5)	ND	ND^a^	ND	0.01 ± 0^a^	0.01	0.01 ± 0^a^
Osbond acid (22:5w6)	ND	0.01 ± 0^a^	ND	0.1 ± 0.01^b^	0.01	0.01 ± 0^a^
α-Linolenic acid (alpha18:3)	ND	0.1 ± 0.02^a^	ND	0.1 ± 0.01^a^	0.8	1.8 ± 0.3^b^
γ-linolenic acid (gamma18:3)	0.02	0.03 ± 0^a^	ND	0.08 ± 0.02^a^	0.1	0.3 ± 0.2^a^

*L. johnsonii* N6.2 were grown to mid-log phase in MRS-TD, MRS-E, and MRS-O, and fatty acids were extracted and quantified by LC–MS. FA Media controls Tween 80 (CT), EA (CE) and OA (CO) were also quantified. Relative concentration is expressed as percentages ± standard deviation of total fatty acids. The quantifications were performed in triplicate and were compared between MRS-TD, MRS-E, and MRS-O by ANOVA and Tukey’s *Post-hoc* test. Different letters denote statistically significant differences of *p* < 0.05. FA which were not detected are denoted ND.

*L. johnsonii* N6.2 in MRS-TD had significantly higher percentages of oleic acid (18:1) and phytomonic acid compared to both MRS-E and MRS-O (*p* < 0.05). Palmitoleic acid (16:1) and linolenic acid (18:2) were significantly higher in MRS-O compared to MRS-TD (*p* < 0.05) (Table 1). The saturated FA of cells grown in MRS-E were significantly different in the percentages of lauric acid (12:0) (1.4 ± 0.1%, *p* < 0.0001) and arachidic acid (20:0) (1.1 ± 0.1%, *p* < 0.0001) when compared to MRS-O (Table 1). Cells grown in MRS-E showed a higher amount of EA (22:1) 25%, (*p* < 0.0001). Interestingly, a significant concentration of nervonic acid (24:1) (3.5 ± 0.7%, *p* < 0.0001), gadoleic acid (20:1 n-11) (0.7 ± 0.1%, *p* < 0.0001), and gondoic acid (20:1 n-9) (6.8 ± 0.6% *p* < 0.0001) were also observed when cells used EA as the only source of FA in the media (Table 1).

### *L. johnsonii* N6.2 modifies EA to generate unique fatty acids

Further analyses of the fatty acid profile of *L. johnsonii* grown in MRS-E, showed the significant enrichment of specific monounsaturated, polyunsaturated, and saturated fatty acids. The saturated fatty acid synthesized in MRS-E were lauric acid (12:0) (1.4 ± 0.1%) and arachidic acid (20:0) (1.1 ± 0.1%). The analysis of monounsaturated fraction indicated that EA (22:1) 25%, was incorporated into the cells while nervonic acid (24:1) 3.5 ± 0.7%, gadoleic acid (20:1 n-11) 0.7 ± 0.1%, and gondoic acid (20:1 n-9) 6.8 ± 0.6% were endogenously synthesized. The polyunsaturated fatty acid synthesized were dihomo-linoleic acid (20:2) 2.2 ± 0.1%, eicosatetraenoic acid (20:4) 1.3 ± 0.1%, docosadienoic acid (22:2) 7.9 ± 0.2%, docosatrienoic acid (22:3) 4 ± 0.2%, and ardenic acid (22:4) 0.1 ± 0%. The fatty acids nervonic acid (24:1) and docosatrienoic acid (22:3) were exclusively detected in cells grown in MRS-E and were not detected in either MRS-O or MRS-TD cells. Except for docosadienoic acid (22:2), each of the fatty acids described were not detected in the EA added to the culture medium. These results suggest endogenous fatty acid modifications utilizing EA as a precursor for diverse FAs incorporated into the membrane.

### *L. johnsonii* N6.2 incorporates exogenous fatty acids rapidly

We designed a rapid shift assay to evaluate the capability of this strain to switch the metabolism depending on the fatty acid supplied. Cells were grown in MRS-O, collected by centrifugation, washed, resuspended in MRS-E, and incubated for 15 min. This assay was referred to as MRS-O- > E. The complementary experiment was also performed with cells grown in MRS-E resuspended in MRS-O and incubated for 15 min at 37°C (MRS-E- > O). To follow the metabolic changes, we used nervonic (24:1), docosadienoic (22:2), and docosatrienoic acids (22:3) as metabolic markers. The change from MRS-O to MRS-E media indicated that EA (22:1) uptake was efficient and nervonic acid, docosadienoic acid, and docosatrienoic acid were rapidly synthesized (Table 2).

**Table 2:** Selected FA in *L. johnsonii* N6.2 grown in MRS-E and MRS-O and soaked in MRS-O (MRS-E- > O) or MRS-E (MRS-O- > E), respectively.

Fatty acids	MRS-TD	MRS-O	MRS-O- > E	MRS-E	MRS-E - > O
Erucic acid (22:1)	0.2 ± 0.1^c^	0.2 ± 0.1^c^	12.3 ± 0.4^d^	25 ± 0.9^a^	9.9 ± 0.9^b^
Nervonic acid (24:1)	ND^c^	ND^c^	0.4 ± 0.03^b^	3.5 ± 0.7^a^	1.4 ± 0.2^b^
Docosadienoic acid (22:2)	0.01 ± 0^c^	0.02 ± 0^c^	3.3 ± 0.1^b^	7.9 ± 0.2^a^	2.7 ± 0.4^b^
Docosatrienoic acid (22:3)	ND ^c^	ND^c^	1.8 ± 0.1^c^	4 ± 0.2^a^	0.2 ± 0.02^c^

Fatty acids which were not detected are denoted ND.

In MRS-O, the percentage of EA (22:1) was 0.2 ± 0.1%. Once incubated in MRS-E for 15 min (MRS-O- > E), EA was rapidly incorporated to reach 9.9 ± 0.9%. Conversely, EA percentages decreased when cells grown in MRS-E were then transferred to MRS-O (MRS-E- > O). These results suggest that both fatty acids are uptaken by the same proteins or that the different proteins used are readily available. Interestingly, nervonic acid (24:1) was quickly synthesized, increasing from 0% to 3.3 ± 0.1% when OA was replaced with EA in the culture media (MRS-O- > E). Docosodienoic (22:2) and Docosatrienoic acid (22:3) were synthesized from EA to reach 3.3 and 1.8% in the MRS-O- > E assay. In summary, nervonic acid production was specifically triggered by the EA since it rapidly decreased once the cells were switched to MRS-O (50% decrease; Table 2). These results were confirmed from the reverse assays, where the quantity of those fatty acids was directly related to the use of EA as a primary source.

### Erucic acid induces the transcription of genes involved in fatty acid metabolism

RNASeq analysis was employed to assess transcriptional changes induced by EA. The transcriptional level of each gene determined in MRS-TD was used as a control to estimate differences in expression. A total of 695 genes showed significant differences in expression with a *p*-adj < 0.05. It was found that 384 were induced while 311 were repressed. The genes with the highest differences in relative expression included 32 ABC transporters, 27 hydrolases, 11 permeases, and 64 hypothetical proteins (Supplementary Table S2).

The large amount of differentially expressed genes may be in part attributed to the previously observed differences in growth kinetics of *L. johnsonii* N6.2 in MRS-E compared to MRS-TD (Thompson et al., 2023). Pathway analysis did not result in significant differences in specific KEGG categories however, several genes significantly induced belonged to the metabolism of lipids and fatty acids. Three of those genes T285_RS04660, T285_RS04710, and T285_RS05090 encode for hypothetical proteins containing a DegV-domain (see Table 3). This domain is present in a family of proteins containing two different *α*/*β* folds associated with binding fatty acids, usually named FakB. FakB is responsible for binding exogenous FAs as they enter the cell for phosphorylation by FakA in the first step of the PlsX/PlsY pathway. *Streptococcus pneumoniae* encodes for three FakB proteins that have been determined to preferentially bind saturated FAs (FakB1), monounsaturated FAs (FakB2), and polyunsaturated FAs (FakB3) (Gullett et al., 2019). *L. johnsonii* N6.2 encodes for four FakB proteins. Consequently, *L. johnsonii’s* homologs were named based on the protein sequence identity to the *S. pneumoniae* FakB characterized homologs as follows: T285_ RS04660 (named FakB1 with 31.91% identity to *S. pneumoniae* FakB1), T285_RS05090 (named FakB2 with 31% identity to *S. pneumoniae* FakB2), T285_RS00120 (named FakB3 with 43% identity to *S. pneumoniae* FakB3) and T285_ RS04710 (named FakB4 with 31.91% identity to *S. pneumoniae* FakB1) (Beliakoff et al., 2024).

**Table 3:** Significantly up- and down-regulated genes in *L. johnsonii* N6.2 grown in MRS-E in the KEGG term lipid biosynthesis.

Locus tag	Gene name	log2	*p*-adj
T285_RS04525	*rbsD*	3.00	2.17E-11
T285_RS04630	*recU*	2.62	1.13E-54
T285_RS04295	*ppc*	2.35	2.89E-73
T285_RS05720	*mreD*	2.22	0.01
T285_RS04080	*ybeY*	2.06	6.05E-31
T285_RS04710	*fakB4*	1.87	1.91E-15
T285_RS04660	*fakB1*	1.84	1.02E-52
T285_RS03990	*prmA*	1.69	2.01E-15
T285_RS04755	*parC*	1.36	7.98E-27
T285_RS03880	*eno3*	1.29	5.40E-05
T285_RS04570	*mvk*	1.25	1.23E-12
T285_RS04015	*aspS*	1.18	9.34E-11
T285_RS04765	*plsY2*	1.18	0.005
T285_RS03135	*galU*	1.08	3.34E-09
T285_RS04565	*mvaD*	1.08	1.74E-10
T285_RS03725	*rseP*	1.08	7.34E-09
T285_RS04595	*asnS*	1.07	1.22E-05
T285_RS04605	*nth*	1.06	0.007
T285_RS04260	*pyrR*	1.06	0.01
T285_RS03070	*hpt*	1.05	4.52E-23
T285_RS06180	*eno1*	1.04	1.63E-47
T285_RS01135	*pgmB*	1.03	0.0009
T285_RS02505	*hpt*	1.01	0.0003
T285_RS04360	*eno2*	0.97	0.004
T285_RS03685	*plsC3*	0.97	0.002
T285_RS03770	*truB*	0.97	2.76E-15
T285_RS02730	*psd*	0.74	2.11E-07
T285_RS03540	*plsX*	0.72	0.0005
T285_RS02740	*psd*	0.55	5.69E-05
T285_RS03840	*cls*	0.44	0.01
T285_RS06380	*pgsA*	0.32	0.08*
T285_RS05090	*fakB2*	0.31	0.031
T285_RS03680	*pmtA*	0.30	0.054*
T285_RS06440	*oleate hydratase* (*OH*)	−0.52	4.26E-07
T285_RS01370	*acpS*	−1.02	0.0008

^*^Not significant but tested in this report. RNASeq analysis was performed on *L. johnsonii* N6.2 grown in MRS-E and compared to standard MRS-TD. Upregulated genes had a *p*-adj < 0.05 and a log2 Fold Change >0 while downregulated genes had a *p*-adj < 0.05 and log2 Fold Change <0.

The T285_RS03530 gene encoding for a putative a DAK2 domain containing protein (FakA) was expressed at high levels in all the conditions, indicating that the *fakA* is constitutively expressed. In agreement with these data, several genes belonging to the PlsX/PlsY pathway were also induced in MRS-E. These included phosphate acyltransferase *plsX;*, glycerol-3-phosphate 1-O-acyltransferase *plsY2*, and 1-acyl-sn-glycerol-3-phosphate acyltransferase *plsC3* (Table 3). Likewise, the transcription of genes encoding enzymes involved in lipid head group biosynthesis were significantly affected when cells were grown in MRS-E. These included a significant upregulation of phosphotidylserine decarboxylases involved in the synthesis of phosphatidylethanolamine T285_RS02730 (*psd1*) and T285_RS02740 (*psd2*) (Table 3).

Other highly transcribed genes upregulated in MRS-E unrelated to fatty acid or lipid metabolism were *rbsD*, *recU*, *ppc*, *mreD*, and *ybeY*. However, in this report, we will focus on the analysis of FA incorporation and integration into membrane phospholipids.

### EA induces alternative pathways for FA incorporation in *Lactobacillus johnsonii* N6.2

Based on the dynamic transformations between FA when cells were transferred between MRS-E and MRS-O, we hypothesized that many components of the FA uptake and utilization pathways might be differentially induced depending on the exogenous availability of FA. *In silico* analyses were performed to identify homologs of the genes identified in the RNASeq analysis involved in FA incorporation. The genes identified were T285_RS00925 (*plsY1*) encoding a glycerol-3-phosphate 1-O-acyltransferase, and three ecoding 1-acyl-sn-glycerol-3-phosphate acyltransferases T285_RS00335 (*plsC2*); T285_RS00100 (*plsC1*) and T285_RS00315 (*plsC4*). However, while all these genes were expressed in both conditions, no significant differences of expression between the MRS-TD and MRS-E were observed. To determine whether a differential increased expression might explain the observed results, a new assay was performed. *L. johnsonii* N6.2 was grown to mid-log phase in MRS-TD, quickly collected by centrifugation and washed once with MRS-NT. Cells were resuspended in either MRS-E, MRS-O or MRS-TD and samples were collected at different time points (5, 15, 30, and 60 min). Gene expression levels were quantified by qRT-PCR and the values obtained are relative to those obtained in MRS-TD after 5 min.

**Figure 1:**
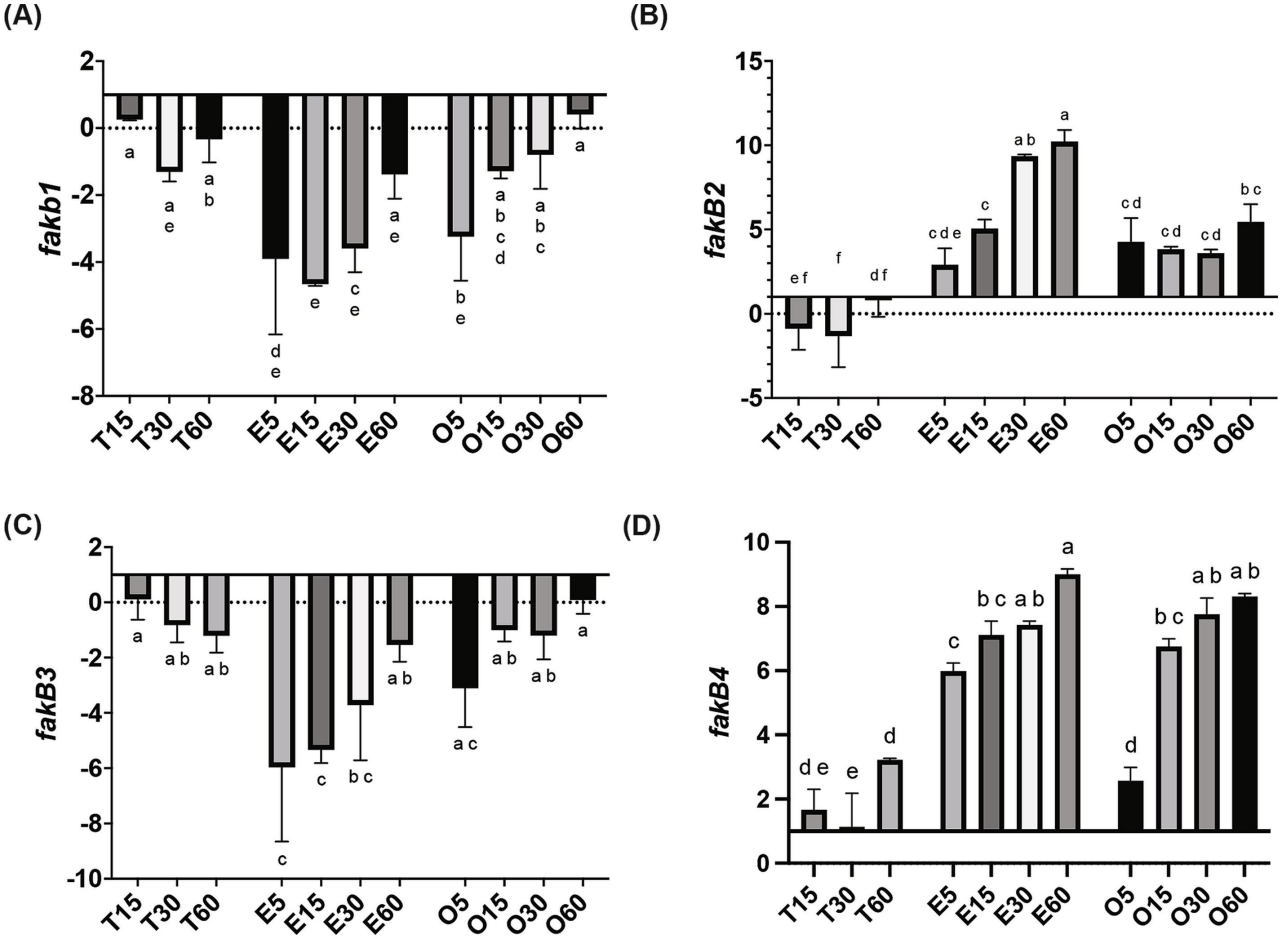
Relative gene expression of fatty acid incorporation genes in *L. johnsonii* N6.2. Cells were grown in MRS-TD to exponential phase, centrifuged and washed pellets were resuspended in either MRS-E (E), MRS-O (O), or MRS-TD (T) for 5, 15, 30, and 60 min. Gene expression is expressed as log(2) fold change relative to MRS-TD 5 min. Genes involved in incorporation of fatty acids included (A) *fakB1*, (B) *fakB2*, (C) *fakB3*, and (D) *fakB4*. Gene expression was normalized to *rpoD*. Statistical analyses were performed by two-way ANOVA and Tukey’s *post-hoc* test. Different letters denote significant difference of *p*-adj < 0.05.

**Figure 2:**
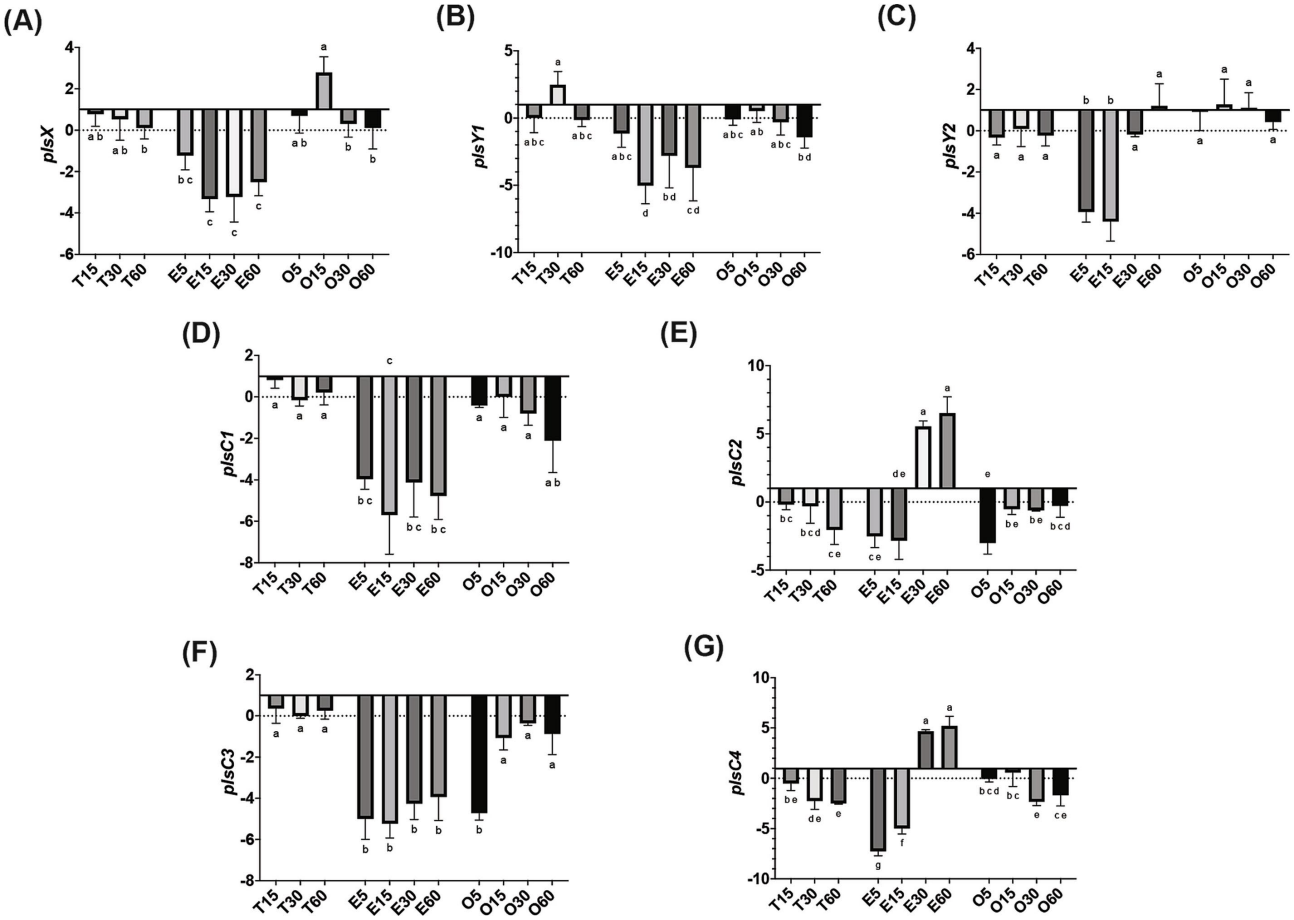
Relative gene expression of phosphatidic acid biosynthesis genes in *L. johnsonii* N6.2. Cells were grown in MRS-TD to exponential phase, centrifuged and washed pellets were resuspended in either MRS-E (E), MRS-O (O), or MRS-TD (T) for 5, 15, 30, and 60 min. Gene expression is expressed as log(2) fold change relative to MRS-TD 5 min. Phosphatidic biosynthesis genes included (A) *plsX*, (B) *plsY1*, (C) *plsY2*, (D) *plsC1*, (E) *plsC2*, (F) *plsC3*, and (G) *plsC4*. Gene expression was normalized to *rpoD*. Statistical analyses were performed by two-way ANOVA and Tukey’s *post-hoc* test. Different letters denote significant difference of *p*-adj < 0.05.

**Figure 3:**
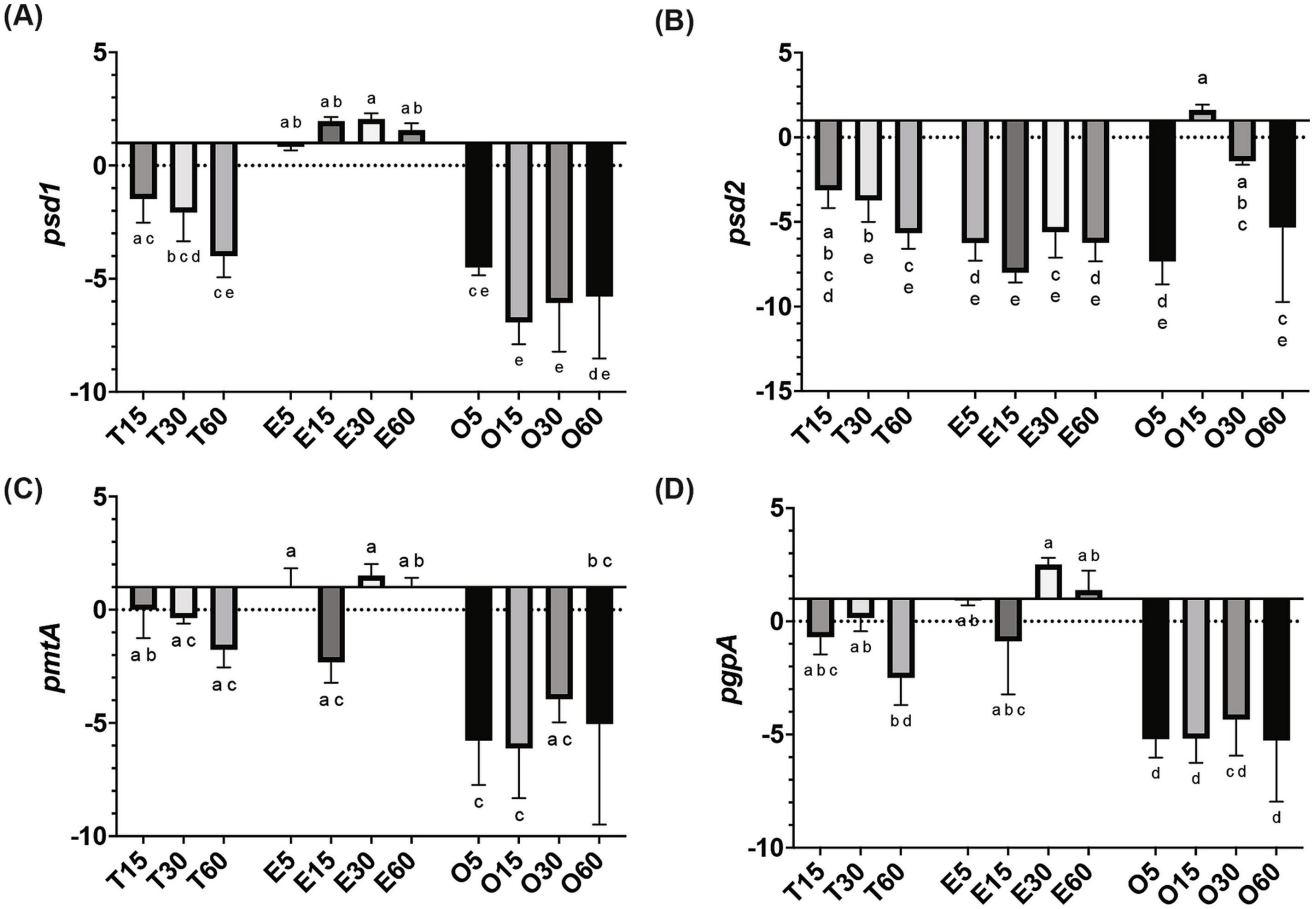
Relative gene expression of lipid head group biosynthesis genes in *L. johnsonii* N6.2. Cells were grown in MRS-TD to exponential phase, centrifuged and washed pellets were resuspended in either MRS-E (E), MRS-O (O), or MRS-TD (T) for 5, 15, 30, and 60 min. Gene expression is expressed as log(2) fold change relative to MRS-TD 5 min. Lipid head group biosynthesis (A) *psd1*, (B) *psd2*, (C) *pmtA*, and (D) *pgpA* were evaluated. Gene expression was normalized to *rpoD*. Statistical analyses were performed by two-way ANOVA and Tukey’s *post-hoc* test. Different letters denote significant difference of *p*-adj < 0.05.

In MRS-E, *fakB1* and *fakB3* expression were significantly downregulated compared to MRS-TD after 15 min (Figures 1A–C). Conversely, EA induced the expression of *fakB2* and *fakB4* (Figures 1B–D), while only *fakB4* was significantly induced in MRS-O. The gene *plsX* encoding an acyl-ACP/phosphate transacylase was differentially induced in in MRS-O while it was not modified in MRS-E or MRS-TD (Figure 2A). The genes encoding sn-1-fatty acyl transferase (*plsY1 and plsY2*) were non significatively induced (Figure 2C). Conversely, *plsC2* and *plsC4* were highly induced in the presence of MRS-E, while *plsC1* and *plsC3* were highly expressed only MRS-TD (Figures 2D–G). It was also found that the expression of genes encoding phosphotidylglycerophosphatase A (*pgpA*) and a phosphatidylserine decarboxylase 1 (*psd1*) were also significantly induced with MRS-E (Figure 3). These results suggest that the changes in the fatty acids pool might also affect the lipid composition of the cell however, the impact on membrane composition needs to be further investigated.

## Discussion

**Figure 4:**
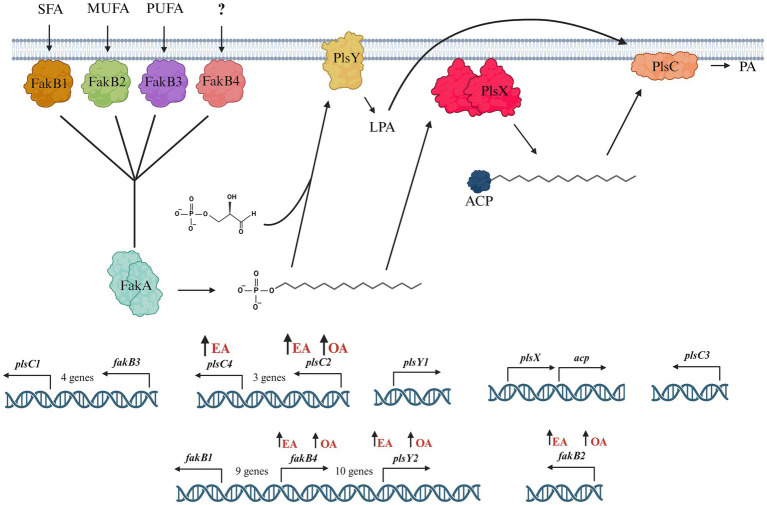
Model of the phosphatidic acid biosynthesis pathway in *L. johnsonii* N6.2. Exogenous fatty acids, saturated fatty acids (SFA), monounsaturated fatty acids (MUFA), and polyunsaturated fatty acids (PUFA) are bound by FakB and converted to acyl-phosphate by FakA. *L. johnsonii* N6.2 encodes four *fakB* genes: *fakB1*, *fakB2*, *fakB3*, and *fakB4*. *fakB2* and *fakB4* are both upregulated in MRS-E media. In *L. johnsonii* N6.2, *plsX* is constitutively expressed in the short term exposure experiments, and modification of fatty acids is likely mediated through alternate enzymes. Acyl-phosphate can be utilized by PlsY which catalyzes the formation of lysophosphatidic acid, or by PlsX to synthesize acyl-ACP. *L. johnsonii* N6.2 encodes two *plsY* genes. *PlsY2* is upregulated in both MRS-E and MRS-O media. Finally, lysophosphatidic acid and acyl-ACP are converted to phosphatidic acid by *PlsC*. *L. johnsonii* N6.2 encodes four *plsC* genes. *PlsC2* and *plsC4* are both upregulated in MRS-E media. Upregulation of the genes by the addition of EA or OA is indicated with an upward arrow next to the FA. Figure created with Biorender.com.

In this study, we investigated the pathways involved in uptake and incorporation of exogenous long chain fatty acids in *L. johnsonii* N6.2. Three general steps are involved in bacteria fatty acid biosynthesis: initiation, cyclic elongation, and termination. The FASII pathway, which occurs in most Gram + and Gram – bacteria, starts with the condensation of Malonyl-CoA and the acyl carrier protein (ACP) to form the Malonyl-ACP. Subsequently, an arrangement of independent enzymes performs a series of reiterative and successive cycles to generate a myriad of fatty acids. The large subset of enzymes and the ACPs necessary to synthesize all the necessary fatty acids (saturated, unsaturated) demand large amounts of energy. Thus, the transcription of the genes involved is tightly regulated. The arrangement these genes as clusters in *S. pneumoniae* facilitates the regulation, while the scattered distribution of individual genes in *Escherichia coli* makes the regulation more complex (My et al., 2013). However, both systems immediately shut down the expression of biosynthetic enzymes upon the availability of fatty acids in the environment. In *S. pneumoniae*, the availability of fatty acids, particularly long-chain acyl-ACP, directly affects the regulation of fatty acid metabolic genes. Acyl-ACP binds to the FabT repressor, increasing its affinity for the DNA sequences controlling the fatty acid biosynthesis genes. When long-chain acyl-ACP levels are high, it promotes stronger repression of these genes, thereby reducing fatty acid production. This creates a feedback loop where the end products of fatty acid synthesis (such as acyl-ACP) inhibit further fatty acid gene expression based on cellular needs (Jerga and Rock, 2009). In contrast, in microorganisms thriving in biological niches where fatty acids are readily available, most genes of the FASII pathways are absent. In *L. johnsonii* N6.2 all the genes encoding for the biosynthetic FAS II pathway are missing, except for FabG and Oar1 (3-oxoacyl-ACP reductases). Consequently, this bacterium depends strictly on fatty acids assimilated from the environment for growth. The FakA/FakB system is highly conserved in Firmicutes and mediates the incorporation of exogenous fatty acids (see Figure 4). In *L. johnsonii* N6.2, while FakA showed constitutive expression, four FakB gene homologs were identified *in silico*. These four proteins have the characteristic DegV domain. This canonical *α*/*β* fold domain consists of seven β-strands and six α-helices to bind fatty acids. The encoded proteins showed sequence identity with their respective *S. pneumoniae* homologs. In *S. pneumoniae*, FakB1 preferentially binds SFA, FakB2 binds MUFAs, while FakB3 is more promiscuous proteins binding SFA, MUFA, and PUFAs (Gullett et al., 2019). However, *S. pneumoniae* only encodes three FakB, while *L. johnsonii* N6.2 encodes four (named here FakB4). We hypothesize that FakB4 may serve to expand the bacterium’s ability to incorporate diverse, host-derived, dietary FAs present in the gut. Our results suggest that *L. johnsonii* FakB proteins are differentially induced depending on the FA sources present in the culture media. Interestingly, as the rapid fatty acid shift assays demonstrated, this induction may also depend on the acyl chain’s length. FakB2 is strongly induced by EA while both OA and EA induced FakB4. In contrast, FakB1 behaved differently between the RNAseq data and the short term exposure to EA. Based on the predicted preference of FakB1 for saturated FA, these results suggest that the induction observed in the RNAseq in MRS-E maybe explained as a response to cellular stress or to the presence of saturated FA in the media.

After binding by FakB and phosphorylation by FakA, the fatty acids are transformed into acyl-ACPs by PlsX. *L. johnsonii* N6.2 encodes only one ACP, which has a high constitutive level of expression in *L. johnsonii* N6.2. These results suggest that incorporation of fatty acids is transcriptionally regulated before and after the acyl-ACP biosynthesis. The presence of multiple *plsY* and *plsC* homologs in *L. johnsonii* N6.2 led us to hypothesize that specific homologs are involved in the incorporation of different FA into phosphatidic acid. In fact, *plsY2*, *plsC2*, and *plsC4* were specifically induced by EA. *Shewanella livingstonensis* Ac10 and *Neisseria meningitidis* have been shown to encode for multiple *plsC* homologs (Toyotake et al., 2018; Shih et al., 1999). In *N. meningitidis*, the two *plsC* homologues, *nlaA* and *nlaB*, possess different activity. In *nlaA* deletion mutants, cells accumulated lysophosphatidic acid (LPA) and retain LPA acyltransferase activity. In contrast, *nlaB* deletion mutants displayed the complementary phenotype by exhibiting decreased LPA acyltransferase activity but did not accumulate LPA (Shih et al., 1999). *S. livingstonensis* Ac10 encodes for five *plsC* homolog. Of those, *PlsC1* of *S. livingstonensis* is involved in the formation of lipids containing eicosopentaenyl groups while *plsC4* is involved in the incorporation of 13:0 and 15:0 fatty acids (Toyotake et al., 2018). Given this data, we propose that that *plsC2* and *plsC4* may be involved in the incorporation of EA (22:1). Further studies are needed to confirm their specific activity. Finally, in *L. johnsonii* N6.2, LPA is later converted into phosphatidylcholines and cardiolipins as it suggested by changes in the expression of the *psd*, *pgsA*, and *cls* genes depending on the FA used.

Since the canonical FA biosynthetic and elongation pathways are missing in *L. johnsonii* N6.2, it is uncertain how the pathways found would allow *L. johnsonii* N6.2 to synthesize saturated and unsaturated fatty acids from EA or OA. Specifically, the biosynthesis of significant amounts of MUFAs like nervonic, gadoleic, and gondoic acids using EA as a source. While it is not yet clear the mechanism of elongation of these fatty acids, we hypothesize that alternative pathways, such as a non-canonical elongation pathway may be facilitating the extension of fatty acid chains. This mechanism could involve the use of metabolites synthesized during the early steps of the isoprenoid biosynthesis pathway like mevalonate. The RNAseq analysis suggest that there is an increase in the glycolysis carbon flow from phosphoenol pyruvate toward oxalacetate. This metabolic bypass would subsequently increase the intracellular pool of acetyl-CoA and acetoacetyl-CoA. These two metabolites can be condensed into hydroxyethyl-glutaryl-CoA, initiating isoprenoid biosynthesis. The induction of genes encoding a mevalonate kinase (*T285_RS04570*) and a phosphomevalonate decarboxylase (*T285_RS04565*) in MRS-E support this hypothesis (Table 3). Acetyl-CoA is also necessary to synthesize fatty acids with longer acyl chains. Thus, part of the acyl-ACP synthesized by PslX is used to synthesize various fatty acids detected in the cells. This could represent a defensive mechanism to achieve acyl-ACP homeostasis because the accumulation of acyl-ACPs causes feedback inhibition on biosynthetic enzymes (Heath and Rock, 1995; Dubnau and Davidoff-Abelson, 1971). The diversification of the fatty acids could be a consequence of the pool of enolases induced in MRS-E (*eno1*, *eno2*, and *eno3*) (Table 3). In *Lactiplantibacillus plantarum*, enolases are multirole enzymes involved in the biohydrogenation and isomerization of long-chain PUFA. The production of a subset of hydroxy fatty acids, oxo fatty acids, conjugated fatty acids, and partially saturated trans-fatty acids was also described in *L. acidophilus* (Ortega-Anaya and Hernández-Santoyo, 2015).

The enrichment of health-relevant FA observed when by *L. johnsonii* N6.2 is grown in EA could have important implications for the strain’s probiotic capabilities. We observed that two nutritionally important omega-9 fatty acids: nervonic and gondoic acid were synthesized from EA. These results indicate that the presumed toxicity of EA present in human consumable oils (i.e., canola oil) could be mitigated by *L. johnsonii* N6.2. The administration of this probiotic could potentially help in the detoxification process in the gastrointestinal tract while increasing the bioavailability of exogenous nervonic acid. In humans, nervonic acid (24:1n-9) plays a significant role as an essential component of shingolipids and it is the rate-limiting step in myelin sheath (Lewkowicz et al., 2019). However, *de novo* synthesis of nervonic acid in hepatocytes and adipocytes does not fulfill the physiological requirements. Recently, this fatty acid has become of distinct interest due to its implications in neurological health and neurodegenerative diseases. The administration of nervonic acid has been shown to improve motor disorder symptoms in murine models of Parkinson’s disease, and it has been indicated as a possible treatment in adrenoleukodystrophy (Hu et al., 2021; Terluk et al., 2022). Nervonic acid has also been shown to have immunomodulatory effects. For example, in a murine model of colitis, nervonic acid was shown to restore the intestinal barrier and suppress NF-kB signaling pathways (Yuan et al., 2023). Nutritional interventions using oils enriched in nervonic acid drastically improved cognitive impairments via direct acceleration of neuronal remyelination. Lipidomic analysis of brain samples extracted from animals after 7 days of treatment showed a significant increase in sphingolipids while the concentrations of glycerolipids decreased significantly (Song et al., 2022). These results demonstrate that nervonic acid could be assimilated at the gastrointestinal level. Dietary EA, in combination with *L. johnsonii* N6.2, could be used as a viable strategy to mitigate, in the long term, cognitive impairment. While scarce information is to discuss the health significance of gondoic and gadoleic acids, a recent report suggested that gondoic acid inhibited the expression of NLRP3 and IL-1β in LPS-exposed liver macrophages. These results were correlated with a decreased expression and secretion of the proinflammatory cytokines IL-1alpha and IL-6 (Fan et al., 2022).

In summary, *L. johnsonii* is able to assimilate exogenous long-chain fatty acids using the FakA/B system as well specific PlsY and PlsC homologs to fulfill its FA. Moreover, EA could efficiently be used as a unique amendment of the MRS to obtain bacterial biomass. The metabolic transformation of EA into health-relevant omega-9 fatty acids would add a significant trait to the strain’s probiotic capabilities. Understanding the mechanisms of fatty acid metabolism in gut bacteria like *L. johnsonii* N6.2 provides valuable insights into how these microbes influence human health and the efficacy of pharmaceuticals. The interactions between bacterial lipid metabolism and host physiology could impact drug absorption, bioavailability, and therapeutic outcomes. By elucidating these pathways, future research may reveal how to optimize the formulation and effectiveness of treatments, particularly in areas related to metabolic disorders, neurological health, and inflammation. Further investigation should be undertaken to elucidate the effects of dietary lipids on the beneficial effects of probiotics such as *L. johnsonii* N6.2, and how these results may translate to the application of probiotics *in vivo*.

## Data Availability

The datasets presented in this study can be found in the NCBI Sequence Read Archive online repository. The BioProject number is PRJNA1137922. https://www.ncbi.nlm.nih.gov/bioproject/PRJNA1137922.
