# Supplementary material for: Erucic acid utilization by Lactobacillus johnsonii N6.2
Source: Front Microbiol. 2024 Nov 25;15:1476958. doi: 10.3389/fmicb.2024.1476958 (PMC11625735; doi:10.3389/fmicb.2024.1476958)
Supplement: Supplementary file 1 [file Table_1.docx]

**Table S1:** Primer sequences used for qRT-PCR analysis.

| Locus | Gene ID | Forward | Reverse |
| --- | --- | --- | --- |
| T285_RS00100 | *plsC1* | TGCTGCTTTCTTTTGGAGGT | GAAAACCTCGTCCTCTGCAA |
| T285_RS00335 | *plsC2* | GAGGTCTACCAGTCGGCAAA | CTTGCCAAATTTGCGTTTTT |
| T285_RS03685 | *plsC3* | AAGGACCAGGATTATCGCGA | TAGCCCCTCATCGAACTTGG |
| T285_RS00315 | *plsC4* | CGGCCCTTTAGCTCCTTTGTT | GCACGCTAAAGTTGAATTGGG |
| T285_RS04660 | *fakB1* | ACCTACGAAACTAGCGGCAT | TGGTCGACTAGTCAAGGCTG |
| T285_RS05090 | *fakB2* | GTCCAAGACGTCCACCCTTA | CGGGACAATTGAAGCTGCTA |
| T285_RS00120 | *fakB3* | CGGCCGTTGTTACCTAAGTT | CGTTCAGCTGGTCCACAATT |
| T285_RS04710 | *fakB4* | TTCCTAAGACTTCGCAGCCT | CGAACGGTCATTGCTGGATC |
| T285_RS00925 | *plsY1* | AGAGCGGGCTTAATTGCTTT | TCCACACGCTTACCCTTACC |
| T285_RS04765 | *plsY2* | TGTAGCAGGCAGTGCAGTTT | CGCTAGTTGCTACCGCTTTT |
| T285_RS03540 | *plsX* | TCGCGCCAACATCAATAATA | GGTTGTTGCTGCGAACTATG |
| T285_RS02730 | *psd1* | CCATAAGCTGCGTCACACAT | GCTTTTATGGATCCACGCGT |
| T285_RS02740 | *psd2* | TGAGTTGAACCGCCAAAGTG | AGCAAATGACTCTCAGGCCT |
| T285_RS03875 | *pgpA* | GGCCCTTGTCAATGAGAACC | ACTACTACCAGTCCCCATGC |
| T285_RS03680 | *pmtA* | TGAACCAACGTGCTCAAACA | ATATTGGTTGTGGCTGGGGA |
| T285_RS04110 | *rpoD* | GATGAAGGCCGTTGACAAAT | ACGAATTGTTCTCGCTTGGT |
