# Supplementary material for: Erucic acid utilization by Lactobacillus johnsonii N6.2
Source: Front Microbiol. 2024 Nov 25;15:1476958. doi: 10.3389/fmicb.2024.1476958 (PMC11625735; doi:10.3389/fmicb.2024.1476958)
Supplement: Supplementary file 2 [file Table_2.docx]

**Table S2.** RNAseq analysis of *Lactobacillus johnsonii* N6.2 grown in MRS-E and compared to MRS-TD. Significantly upregulated (log_2_ fold change > 1) and downregulated genes (log_2_ fold change < 1) are listed.

| Gene Number | Gene Name | log2FoldChange | p-value |
| --- | --- | --- | --- |
| T285_RS06815 | - | 3.75 | 6.3E-118 |
| T285_RS04295 | ppc | 2.35 | 3.3E-76 |
| T285_RS00150 | - | -2.31 | 3.24E-75 |
| T285_RS07885 | - | -2.07 | 4.36E-68 |
| T285_RS04145 | pepT | 1.65 | 4.28E-59 |
| T285_RS04630 | recU | 2.62 | 3.89E-57 |
| T285_RS09295 | - | -1.43 | 2.04E-55 |
| T285_RS04660 | DegV1 | 1.84 | 4.69E-55 |
| T285_RS04690 | - | 1.58 | 5.59E-55 |
| T285_RS06810 | - | 4.00 | 2.82E-54 |
| T285_RS06180 | eno1 | 1.04 | 1.02E-49 |
| T285_RS04670 | - | 1.90 | 1.98E-42 |
| T285_RS04215 | - | 1.93 | 8.97E-40 |
| T285_RS09005 | - | 1.79 | 5.99E-39 |
| T285_RS04075 | - | 2.04 | 2.85E-35 |
| T285_RS07710 | pepT | -1.04 | 4.27E-35 |
| T285_RS04080 | ybeY | 2.06 | 5.88E-33 |
| T285_RS04680 | - | 1.73 | 8.37E-32 |
| T285_RS04555 | - | 1.56 | 6.42E-31 |
| T285_RS08835 | mnmE | -1.50 | 8.01E-31 |
| T285_RS03700 | - | 1.24 | 1.25E-30 |
| T285_RS04530 | - | 2.42 | 4.25E-30 |
| T285_RS04675 | - | 1.96 | 1.38E-29 |
| T285_RS04105 | - | 1.16 | 2.25E-29 |
| T285_RS04755 | parC | 1.36 | 1.14E-28 |
| T285_RS06805 | - | 3.94 | 2.05E-28 |
| T285_RS02630 | - | -1.13 | 1.53E-27 |
| T285_RS07320 | - | -1.20 | 1.48E-27 |
| T285_RS04645 | - | 1.58 | 2.23E-27 |
| T285_RS07840 | - | -1.49 | 3.95E-26 |
| T285_RS01865 | - | -1.39 | 1.54E-25 |
| T285_RS03070 | hpt | 1.05 | 8.27E-25 |
| T285_RS00750 | - | -0.98 | 1.82E-24 |
| T285_RS08815 | - | -1.05 | 2.82E-24 |
| T285_RS04585 | - | 1.72 | 3E-24 |
| T285_RS04290 | - | 2.15 | 4.32E-23 |
| T285_RS03955 | - | 1.31 | 6.86E-23 |
| T285_RS04430 | - | 2.05 | 1.43E-22 |
| T285_RS04560 | - | 1.35 | 1.71E-22 |
| T285_RS07355 | - | -1.00 | 1.72E-22 |
| T285_RS04210 | - | 1.82 | 3.45E-22 |
| T285_RS06820 | - | 3.56 | 4.43E-22 |
| T285_RS04625 | - | 2.29 | 5.81E-22 |
| T285_RS03920 | - | 1.88 | 5.69E-22 |
| T285_RS03940 | - | 1.55 | 1.02E-21 |
| T285_RS04445 | - | 1.49 | 4.34E-21 |
| T285_RS03665 | - | 0.71 | 5.66E-21 |
| T285_RS04300 | - | 1.67 | 9.45E-21 |
| T285_RS02060 | - | 1.20 | 1.93E-20 |
| T285_RS07695 | - | -0.96 | 1.9E-20 |
| T285_RS06765 | thrS | 0.98 | 5.72E-20 |
| T285_RS05250 | uvrC | -1.00 | 5.77E-20 |
| T285_RS06500 | - | -1.47 | 8.19E-20 |
| T285_RS06250 | uvrA | -1.29 | 1.25E-19 |
| T285_RS03775 | ribF | 0.93 | 1.74E-19 |
| T285_RS06185 | - | 1.02 | 3.37E-19 |
| T285_RS01275 | - | -1.77 | 7.25E-19 |
| T285_RS04760 | parE | 1.13 | 1.19E-18 |
| T285_RS04030 | - | 1.10 | 1.72E-18 |
| T285_RS07675 | - | -1.15 | 1.89E-18 |
| T285_RS04725 | xerS | 1.32 | 2.76E-18 |
| T285_RS05705 | rsmH | 0.83 | 3.63E-18 |
| T285_RS04720 | - | 1.15 | 4.55E-18 |
| T285_RS04685 | - | 1.80 | 5.02E-18 |
| T285_RS08930 | - | -2.53 | 7.35E-18 |
| T285_RS07810 | - | -1.27 | 1.06E-17 |
| T285_RS01270 | - | -2.05 | 1.27E-17 |
| T285_RS04575 | - | 1.70 | 1.53E-17 |
| T285_RS06800 | - | 3.74 | 3.43E-17 |
| T285_RS04710 | DegV4(R2) | 1.87 | 7.65E-17 |
| T285_RS08375 | - | -1.10 | 8.04E-17 |
| T285_RS03990 | prmA | 1.69 | 8.38E-17 |
| T285_RS04000 | - | 1.13 | 8.33E-17 |
| T285_RS04305 | - | 2.34 | 1.03E-16 |
| T285_RS03770 | truB | 0.97 | 1.18E-16 |
| T285_RS04060 | - | 1.61 | 1.22E-16 |
| T285_RS04580 | addA | 1.46 | 1.92E-16 |
| T285_RS04700 | - | 1.43 | 3.93E-16 |
| T285_RS04255 | - | 1.97 | 6.6E-16 |
| T285_RS03860 | - | 1.28 | 6.54E-16 |
| T285_RS04340 | - | 1.42 | 9.65E-16 |
| T285_RS04440 | - | 1.75 | 1.84E-15 |
| T285_RS02175 | groL | -0.64 | 2.33E-15 |
| T285_RS03950 | - | 1.04 | 9.82E-15 |
| T285_RS07745 | - | -1.25 | 1.64E-14 |
| T285_RS05845 | atpD | 1.22 | 1.89E-14 |
| T285_RS07845 | - | -0.96 | 2.49E-14 |
| T285_RS01200 | - | -1.13 | 3.83E-14 |
| T285_RS04110 | rpoD | 1.18 | 5.33E-14 |
| T285_RS04235 | - | 1.54 | 5.75E-14 |
| T285_RS04570 | mvk | 1.25 | 6.41E-14 |
| T285_RS06200 | - | -0.92 | 1.3E-13 |
| T285_RS07890 | - | -2.55 | 1.64E-13 |
| T285_RS01185 | - | -0.81 | 1.84E-13 |
| T285_RS05780 | ezrA | 0.92 | 2.52E-13 |
| T285_RS05855 | - | 1.07 | 2.93E-13 |
| T285_RS04310 | - | 1.92 | 3.09E-13 |
| T285_RS04665 | - | 1.61 | 3.42E-13 |
| T285_RS08830 | mnmG | -1.59 | 3.46E-13 |
| T285_RS01140 | ugpC | 1.41 | 5.82E-13 |
| T285_RS04650 | - | 1.30 | 6.08E-13 |
| T285_RS04095 | glyQ | 1.31 | 6.9E-13 |
| T285_RS08775 | - | -0.93 | 7.19E-13 |
| T285_RS08655 | phnD | 1.53 | 7.57E-13 |
| T285_RS02170 | - | -1.39 | 7.69E-13 |
| T285_RS04590 | - | 1.82 | 9.64E-13 |
| T285_RS04715 | - | 1.35 | 1.05E-12 |
| T285_RS04525 | rbsD | 3.00 | 1.34E-12 |
| T285_RS08285 | - | -1.61 | 1.62E-12 |
| T285_RS04100 | - | 1.43 | 1.82E-12 |
| T285_RS04200 | - | 1.27 | 2.19E-12 |
| T285_RS08010 | - | -0.80 | 2.61E-12 |
| T285_RS04230 | - | 2.07 | 2.75E-12 |
| T285_RS01675 | - | -1.03 | 4.09E-12 |
| T285_RS08725 | - | 1.51 | 5.03E-12 |
| T285_RS04015 | aspS | 1.18 | 6.2E-12 |
| T285_RS02985 | phoU | 0.85 | 7.03E-12 |
| T285_RS03590 | ftsY | 0.72 | 7.2E-12 |
| T285_RS06560 | - | -0.82 | 7.66E-12 |
| T285_RS02660 | - | 1.30 | 8.62E-12 |
| T285_RS04565 | mvaD | 1.08 | 1.2E-11 |
| T285_RS08700 | - | -1.26 | 1.99E-11 |
| T285_RS05850 | - | 1.20 | 2.26E-11 |
| T285_RS07915 | hflX | -1.13 | 2.72E-11 |
| T285_RS05155 | - | 1.79 | 2.82E-11 |
| T285_RS03705 | - | 0.71 | 3.49E-11 |
| T285_RS00970 | - | -1.37 | 3.98E-11 |
| T285_RS04170 | - | 1.74 | 4.27E-11 |
| T285_RS01640 | - | -1.31 | 4.43E-11 |
| T285_RS04930 | - | 1.65 | 5.14E-11 |
| T285_RS09075 | ssrA | 1.55 | 5.5E-11 |
| T285_RS03640 | - | 1.22 | 5.57E-11 |
| T285_RS09015 | - | 1.11 | 9.69E-11 |
| T285_RS08605 | - | -0.75 | 1.12E-10 |
| T285_RS03415 | nusB | 1.26 | 1.17E-10 |
| T285_RS01650 | truA | -0.93 | 1.31E-10 |
| T285_RS03960 | - | 1.65 | 1.37E-10 |
| T285_RS00490 | - | 0.73 | 1.38E-10 |
| T285_RS04735 | - | 1.01 | 1.47E-10 |
| T285_RS04315 | - | 1.56 | 1.96E-10 |
| T285_RS04055 | - | 1.17 | 2.03E-10 |
| T285_RS04425 | - | 1.82 | 2.3E-10 |
| T285_RS03135 | galU | 1.08 | 2.73E-10 |
| T285_RS04135 | - | 1.06 | 2.78E-10 |
| T285_RS05330 | - | -0.81 | 2.93E-10 |
| T285_RS08355 | - | -0.86 | 2.91E-10 |
| T285_RS01725 | - | -0.60 | 3.12E-10 |
| T285_RS04190 | - | 1.65 | 3.44E-10 |
| T285_RS08365 | - | 0.65 | 4.06E-10 |
| T285_RS05755 | - | 0.59 | 4.25E-10 |
| T285_RS07555 | serS | 1.16 | 4.32E-10 |
| T285_RS07715 | - | -1.13 | 4.53E-10 |
| T285_RS00675 | phnC | 0.84 | 6.13E-10 |
| T285_RS03405 | efp | 0.90 | 6.25E-10 |
| T285_RS03725 | rseP | 1.08 | 6.5E-10 |
| T285_RS04480 | - | 1.15 | 6.76E-10 |
| T285_RS08460 | - | -0.58 | 7.59E-10 |
| T285_RS03650 | - | 1.18 | 8.85E-10 |
| T285_RS08795 | - | -0.82 | 1.01E-09 |
| T285_RS04220 | - | 1.52 | 1.12E-09 |
| T285_RS04545 | - | 1.80 | 1.4E-09 |
| T285_RS08845 | rnpA | -1.21 | 1.42E-09 |
| T285_RS06150 | - | 0.98 | 1.55E-09 |
| T285_RS03530 | - | 0.59 | 1.7E-09 |
| T285_RS00160 | - | -1.27 | 1.73E-09 |
| T285_RS04750 | - | 2.09 | 1.96E-09 |
| T285_RS05525 | holA | 0.74 | 2.1E-09 |
| T285_RS01130 | - | 1.55 | 2.23E-09 |
| T285_RS09095 | - | -1.53 | 2.34E-09 |
| T285_RS00140 | - | -0.67 | 2.68E-09 |
| T285_RS06705 | - | -0.66 | 2.99E-09 |
| T285_RS02670 | - | 0.91 | 3.01E-09 |
| T285_RS05480 | - | -0.53 | 3.33E-09 |
| T285_RS03660 | - | 0.98 | 3.78E-09 |
| T285_RS00680 | phnE | 0.76 | 3.81E-09 |
| T285_RS06190 | - | 0.84 | 4.14E-09 |
| T285_RS05800 | - | 1.66 | 5.31E-09 |
| T285_RS04795 | - | 1.27 | 5.53E-09 |
| T285_RS04640 | - | 1.79 | 5.76E-09 |
| T285_RS01320 | - | -0.95 | 7.17E-09 |
| T285_RS01465 | - | -0.51 | 7.38E-09 |
| T285_RS02655 | - | 1.37 | 8.07E-09 |
| T285_RS08145 | - | -0.60 | 8.12E-09 |
| T285_RS03715 | - | 1.09 | 9.98E-09 |
| T285_RS01075 | metG | -0.57 | 1.01E-08 |
| T285_RS01435 | hslO | -0.94 | 1.01E-08 |
| T285_RS03570 | - | -1.20 | 1.81E-08 |
| T285_RS02730 | psd | 0.74 | 2.28E-08 |
| T285_RS07160 | - | -0.94 | 2.27E-08 |
| T285_RS08150 | - | -0.65 | 2.88E-08 |
| T285_RS08155 | - | -0.57 | 2.95E-08 |
| T285_RS04070 | - | 1.38 | 3.09E-08 |
| T285_RS00185 | - | -0.87 | 3.34E-08 |
| T285_RS01470 | rpoC | -0.53 | 3.39E-08 |
| T285_RS02015 | rimI | -0.59 | 3.59E-08 |
| T285_RS03440 | - | 0.69 | 4E-08 |
| T285_RS05840 | - | 1.09 | 4.12E-08 |
| T285_RS04655 | - | 2.38 | 4.48E-08 |
| T285_RS06440 | OH | -0.52 | 4.85E-08 |
| T285_RS01000 | - | 1.37 | 5.14E-08 |
| T285_RS03445 | recN | 0.75 | 5.26E-08 |
| T285_RS04370 | - | 3.86 | 5.32E-08 |
| T285_RS03915 | - | 1.54 | 5.54E-08 |
| T285_RS08800 | - | -0.93 | 6E-08 |
| T285_RS02020 | tsaD | -0.53 | 6.22E-08 |
| T285_RS03875 | - | 0.77 | 6.32E-08 |
| T285_RS00460 | - | 0.76 | 7.19E-08 |
| T285_RS00380 | rlmH | -0.87 | 7.77E-08 |
| T285_RS05030 | - | 0.69 | 9.22E-08 |
| T285_RS06640 | - | -0.50 | 1.18E-07 |
| T285_RS07645 | - | 1.21 | 1.23E-07 |
| T285_RS04320 | - | 0.63 | 1.5E-07 |
| T285_RS04050 | sdaAA | 1.17 | 1.53E-07 |
| T285_RS03420 | - | 0.80 | 1.59E-07 |
| T285_RS08825 | spxB | -1.01 | 1.69E-07 |
| T285_RS00505 | - | -0.75 | 1.71E-07 |
| T285_RS05875 | atpB | 0.76 | 1.78E-07 |
| T285_RS06865 | - | -1.20 | 1.78E-07 |
| T285_RS01395 | mfd | -0.43 | 1.92E-07 |
| T285_RS03760 | infB | 0.64 | 2.16E-07 |
| T285_RS02140 | - | -0.49 | 2.35E-07 |
| T285_RS05860 | - | 1.52 | 2.53E-07 |
| T285_RS00465 | - | -1.01 | 2.55E-07 |
| T285_RS04985 | budA | -0.67 | 2.61E-07 |
| T285_RS03435 | - | 0.62 | 2.86E-07 |
| T285_RS00180 | xth | -1.13 | 3.27E-07 |
| T285_RS00755 | - | -0.57 | 3.34E-07 |
| T285_RS04435 | - | 1.61 | 3.4E-07 |
| T285_RS07335 | - | -0.87 | 3.86E-07 |
| T285_RS04005 | - | 1.47 | 4.05E-07 |
| T285_RS04780 | - | 1.28 | 4.17E-07 |
| T285_RS02565 | - | -1.17 | 4.27E-07 |
| T285_RS00510 | - | -0.69 | 4.45E-07 |
| T285_RS06580 | - | 0.57 | 4.6E-07 |
| T285_RS00780 | - | -0.79 | 5.03E-07 |
| T285_RS03635 | - | 1.07 | 5.11E-07 |
| T285_RS03290 | - | 0.69 | 5.48E-07 |
| T285_RS08035 | - | -0.63 | 5.67E-07 |
| T285_RS02775 | - | -0.48 | 6.01E-07 |
| T285_RS01345 | - | 0.71 | 6.05E-07 |
| T285_RS08095 | - | -0.39 | 6.64E-07 |
| T285_RS00745 | - | -0.74 | 6.83E-07 |
| T285_RS00060 | dnaB | -0.48 | 8.2E-07 |
| T285_RS02695 | - | 0.72 | 8.74E-07 |
| T285_RS04185 | - | 1.09 | 8.81E-07 |
| T285_RS03720 | - | 1.31 | 9.21E-07 |
| T285_RS05865 | atpF | 0.98 | 9.43E-07 |
| T285_RS04875 | - | -0.61 | 1.05E-06 |
| T285_RS03820 | - | 0.73 | 1.13E-06 |
| T285_RS00235 | - | -1.48 | 1.15E-06 |
| T285_RS03170 | - | 1.61 | 1.24E-06 |
| T285_RS05500 | tuf | 0.93 | 1.26E-06 |
| T285_RS00660 | - | -0.76 | 1.33E-06 |
| T285_RS00005 | dnaA | -0.89 | 1.37E-06 |
| T285_RS08235 | - | -0.77 | 1.43E-06 |
| T285_RS04090 | recO | 0.78 | 1.56E-06 |
| T285_RS06860 | - | -0.88 | 1.74E-06 |
| T285_RS04595 | asnS | 1.07 | 1.8E-06 |
| T285_RS04860 | - | 1.08 | 2.17E-06 |
| T285_RS04635 | - | 1.97 | 2.39E-06 |
| T285_RS04020 | - | 1.02 | 2.42E-06 |
| T285_RS07775 | - | -1.22 | 2.62E-06 |
| T285_RS02510 | - | 0.52 | 2.67E-06 |
| T285_RS01280 | - | -0.98 | 2.76E-06 |
| T285_RS02210 | - | 1.04 | 2.96E-06 |
| T285_RS01460 | - | -0.59 | 3.07E-06 |
| T285_RS05775 | - | -0.86 | 3.19E-06 |
| T285_RS04240 | - | 1.01 | 3.31E-06 |
| T285_RS07950 | nrdI | -0.96 | 3.41E-06 |
| T285_RS04420 | - | 1.41 | 3.46E-06 |
| T285_RS01900 | rplA | 0.41 | 3.52E-06 |
| T285_RS04335 | - | 1.47 | 4.04E-06 |
| T285_RS07640 | - | -0.63 | 4.02E-06 |
| T285_RS01375 | alr | -0.54 | 4.25E-06 |
| T285_RS03925 | - | 2.34 | 4.34E-06 |
| T285_RS03560 | - | 0.80 | 4.67E-06 |
| T285_RS00325 | - | -0.46 | 4.92E-06 |
| T285_RS07450 | - | -1.69 | 5.08E-06 |
| T285_RS02735 | - | 1.35 | 5.13E-06 |
| T285_RS03340 | - | 0.54 | 5.99E-06 |
| T285_RS04125 | - | 0.49 | 6.04E-06 |
| T285_RS01225 | - | -0.57 | 6.23E-06 |
| T285_RS00075 | - | -0.90 | 6.23E-06 |
| T285_RS01635 | - | -0.84 | 6.58E-06 |
| T285_RS03265 | - | 0.73 | 6.64E-06 |
| T285_RS06085 | - | 0.43 | 6.62E-06 |
| T285_RS06220 | - | -0.52 | 6.77E-06 |
| T285_RS07970 | - | -0.81 | 7.1E-06 |
| T285_RS08330 | - | -0.57 | 7.62E-06 |
| T285_RS05645 | - | 0.46 | 8.13E-06 |
| T285_RS06495 | - | -0.66 | 8.44E-06 |
| T285_RS03695 | rpsB | 0.81 | 8.55E-06 |
| T285_RS03755 | - | 0.51 | 8.83E-06 |
| T285_RS03880 | eno3 | 1.29 | 9.07E-06 |
| T285_RS02740 | psd | 0.55 | 9.6E-06 |
| T285_RS04540 | - | 0.79 | 1.18E-05 |
| T285_RS04380 | - | 1.23 | 1.3E-05 |
| T285_RS02190 | - | -0.63 | 1.35E-05 |
| T285_RS07245 | - | -0.45 | 1.4E-05 |
| T285_RS09210 | - | 0.90 | 1.43E-05 |
| T285_RS06260 | - | 0.51 | 1.49E-05 |
| T285_RS06370 | rny | -0.72 | 1.62E-05 |
| T285_RS04705 | - | 1.42 | 1.66E-05 |
| T285_RS02515 | - | -0.92 | 1.69E-05 |
| T285_RS07955 | - | -0.68 | 1.77E-05 |
| T285_RS04600 | - | 1.79 | 1.86E-05 |
| T285_RS08695 | nagB | -0.98 | 1.97E-05 |
| T285_RS00820 | - | 0.77 | 2E-05 |
| T285_RS07960 | - | -0.56 | 2E-05 |
| T285_RS00245 | - | -0.81 | 2.23E-05 |
| T285_RS08715 | - | -0.63 | 2.29E-05 |
| T285_RS02045 | - | 0.86 | 2.41E-05 |
| T285_RS00065 | - | -0.74 | 2.61E-05 |
| T285_RS02560 | - | -1.45 | 2.77E-05 |
| T285_RS07315 | - | -0.86 | 3.07E-05 |
| T285_RS04040 | - | 1.77 | 3.13E-05 |
| T285_RS02650 | - | 0.88 | 3.24E-05 |
| T285_RS00290 | - | -1.28 | 3.48E-05 |
| T285_RS02770 | - | -0.90 | 3.5E-05 |
| T285_RS06920 | - | 0.49 | 3.57E-05 |
| T285_RS05190 | - | -0.47 | 3.56E-05 |
| T285_RS00070 | - | -1.06 | 3.58E-05 |
| T285_RS07945 | - | 0.65 | 3.65E-05 |
| T285_RS03750 | - | 0.49 | 3.68E-05 |
| T285_RS01670 | - | -0.87 | 3.7E-05 |
| T285_RS03535 | recG | 0.49 | 3.78E-05 |
| T285_RS05045 | hslV | 0.58 | 3.81E-05 |
| T285_RS00155 | - | -1.27 | 3.82E-05 |
| T285_RS04505 | - | 1.31 | 4.17E-05 |
| T285_RS03780 | abc-f | 0.57 | 4.49E-05 |
| T285_RS02040 | - | 0.91 | 4.59E-05 |
| T285_RS03655 | - | 1.07 | 4.73E-05 |
| T285_RS01210 | - | -1.40 | 4.98E-05 |
| T285_RS08345 | - | -0.82 | 5.12E-05 |
| T285_RS02675 | - | 0.82 | 5.18E-05 |
| T285_RS04745 | - | 0.85 | 5.23E-05 |
| T285_RS08405 | - | 0.34 | 5.27E-05 |
| T285_RS03165 | - | 0.89 | 5.53E-05 |
| T285_RS08705 | - | -0.74 | 5.99E-05 |
| T285_RS02505 | hpt | 1.01 | 6.11E-05 |
| T285_RS02665 | - | 1.81 | 6.17E-05 |
| T285_RS00450 | - | -0.74 | 6.48E-05 |
| T285_RS03145 | - | 0.88 | 6.51E-05 |
| T285_RS02815 | - | 0.58 | 6.67E-05 |
| T285_RS01910 | - | -0.56 | 6.71E-05 |
| T285_RS08040 | - | -1.00 | 6.82E-05 |
| T285_RS07760 | - | -0.93 | 7.79E-05 |
| T285_RS05750 | - | 0.50 | 8.55E-05 |
| T285_RS00115 | - | -0.99 | 8.83E-05 |
| T285_RS08630 | - | -0.49 | 9.03E-05 |
| T285_RS01290 | - | -0.66 | 9.02E-05 |
| T285_RS04490 | - | 1.20 | 9.23E-05 |
| T285_RS00500 | - | -0.51 | 9.44E-05 |
| T285_RS04515 | - | 1.51 | 9.94E-05 |
| T285_RS03540 | plsX | 0.72 | 0.0001 |
| T285_RS05165 | - | -0.51 | 0.0001 |
| T285_RS05050 | xerC | 0.68 | 0.0001 |
| T285_RS04990 | alsS | -0.74 | 0.0001 |
| T285_RS07670 | - | -0.37 | 0.0001 |
| T285_RS04980 | - | 1.69 | 0.0001 |
| T285_RS07765 | - | -1.82 | 0.0001 |
| T285_RS04415 | - | 2.48 | 0.0001 |
| T285_RS03930 | - | 1.01 | 0.0001 |
| T285_RS03805 | dnaK | 0.84 | 0.0001 |
| T285_RS08045 | - | -0.40 | 0.0001 |
| T285_RS00795 | - | -0.50 | 0.0001 |
| T285_RS07520 | - | -1.93 | 0.0001 |
| T285_RS04085 | - | 0.76 | 0.0001 |
| T285_RS03190 | - | 1.20 | 0.0001 |
| T285_RS07490 | - | -1.38 | 0.0001 |
| T285_RS04785 | - | 1.17 | 0.0001 |
| T285_RS07260 | - | -0.51 | 0.0001 |
| T285_RS09235 | - | 1.21 | 0.0001 |
| T285_RS01890 | nusG | -0.60 | 0.0001 |
| T285_RS06280 | - | -0.77 | 0.0001 |
| T285_RS00190 | - | -0.54 | 0.0001 |
| T285_RS06215 | whiA | -0.45 | 0.0002 |
| T285_RS04150 | - | -0.74 | 0.0002 |
| T285_RS01370 | acpS | -1.02 | 0.0002 |
| T285_RS08000 | - | -0.76 | 0.0002 |
| T285_RS09200 | - | -0.78 | 0.0002 |
| T285_RS07735 | - | -0.51 | 0.0002 |
| T285_RS06360 | - | -1.11 | 0.0002 |
| T285_RS01135 | pgmB | 1.03 | 0.0002 |
| T285_RS03730 | - | 0.83 | 0.0002 |
| T285_RS08350 | - | -0.60 | 0.0002 |
| T285_RS08840 | - | -1.27 | 0.0002 |
| T285_RS04375 | - | 0.93 | 0.0002 |
| T285_RS04550 | - | 0.92 | 0.0003 |
| T285_RS00800 | - | -0.39 | 0.0003 |
| T285_RS06310 | trxB | -0.73 | 0.0003 |
| T285_RS01615 | rpsM | -0.31 | 0.0003 |
| T285_RS07425 | - | -1.88 | 0.0003 |
| T285_RS07420 | - | -1.89 | 0.0003 |
| T285_RS04880 | - | -0.58 | 0.0003 |
| T285_RS01195 | - | -0.72 | 0.0003 |
| T285_RS07235 | - | -1.63 | 0.0003 |
| T285_RS05660 | - | 0.51 | 0.0003 |
| T285_RS03510 | rpe | 0.51 | 0.0003 |
| T285_RS08165 | - | -0.34 | 0.0003 |
| T285_RS04140 | - | 1.04 | 0.0003 |
| T285_RS07965 | - | -0.71 | 0.0003 |
| T285_RS05870 | atpE | 1.12 | 0.0003 |
| T285_RS03010 | rpiA | 0.59 | 0.0004 |
| T285_RS03710 | frr | 1.29 | 0.0004 |
| T285_RS03410 | - | 1.89 | 0.0004 |
| T285_RS01230 | - | -0.45 | 0.0004 |
| T285_RS03585 | smc | 0.43 | 0.0004 |
| T285_RS07030 | - | -0.59 | 0.0004 |
| T285_RS07740 | - | -1.41 | 0.0004 |
| T285_RS02010 | tsaB | -0.49 | 0.0004 |
| T285_RS01190 | - | -0.82 | 0.0005 |
| T285_RS08115 | - | -0.72 | 0.0005 |
| T285_RS03685 | PlsC3 | 0.97 | 0.0005 |
| T285_RS03815 | lepA | 0.36 | 0.0005 |
| T285_RS03745 | nusA | 0.41 | 0.0005 |
| T285_RS03130 | - | 1.34 | 0.0005 |
| T285_RS00265 | - | -0.74 | 0.0005 |
| T285_RS08060 | - | -0.42 | 0.0005 |
| T285_RS01150 | - | 0.95 | 0.0006 |
| T285_RS00875 | - | -0.69 | 0.0006 |
| T285_RS01005 | - | 0.63 | 0.0006 |
| T285_RS02965 | pstC | 0.81 | 0.0006 |
| T285_RS04360 | eno2 | 0.97 | 0.0006 |
| T285_RS00050 | - | -0.52 | 0.0007 |
| T285_RS03995 | - | 2.08 | 0.0007 |
| T285_RS08610 | - | -1.11 | 0.0007 |
| T285_RS00225 | - | -0.58 | 0.0007 |
| T285_RS07130 | - | -0.38 | 0.0007 |
| T285_RS00415 | - | -0.39 | 0.0007 |
| T285_RS07805 | efp | -0.99 | 0.0007 |
| T285_RS03310 | - | 1.17 | 0.0007 |
| T285_RS08100 | - | -0.41 | 0.0007 |
| T285_RS08685 | - | -0.81 | 0.0007 |
| T285_RS01145 | - | 1.01 | 0.0007 |
| T285_RS07325 | - | -0.99 | 0.0008 |
| T285_RS07995 | - | 1.34 | 0.0008 |
| T285_RS04885 | sufB | -0.71 | 0.0008 |
| T285_RS06895 | - | 1.13 | 0.0008 |
| T285_RS06505 | - | -0.91 | 0.0008 |
| T285_RS06080 | - | 1.02 | 0.0008 |
| T285_RS04045 | - | 1.04 | 0.0009 |
| T285_RS01965 | recR | 0.51 | 0.0009 |
| T285_RS00835 | - | -0.42 | 0.0009 |
| T285_RS00110 | - | -0.48 | 0.0009 |
| T285_RS08240 | - | -0.73 | 0.0009 |
| T285_RS04250 | pyrF | 1.10 | 0.0009 |
| T285_RS05825 | - | -0.50 | 0.0009 |
| T285_RS06960 | - | -0.58 | 0.0009 |
| T285_RS05100 | - | 0.51 | 0.001 |
| T285_RS03160 | - | 0.47 | 0.001 |
| T285_RS04870 | - | -0.49 | 0.001 |
| T285_RS08140 | - | 0.40 | 0.001 |
| T285_RS05490 | clpX | -0.38 | 0.001 |
| T285_RS06255 | uvrB | -0.78 | 0.001 |
| T285_RS02605 | - | 1.33 | 0.001 |
| T285_RS04330 | - | 2.26 | 0.001 |
| T285_RS00145 | - | -0.58 | 0.001 |
| T285_RS02260 | alaS | -0.32 | 0.001 |
| T285_RS02330 | - | 1.29 | 0.001 |
| T285_RS04775 | - | 1.18 | 0.001 |
| T285_RS04765 | plsY2 | 1.18 | 0.001 |
| T285_RS07065 | pcrA | -0.33 | 0.001 |
| T285_RS04800 | - | 0.70 | 0.001 |
| T285_RS08185 | - | -0.28 | 0.001 |
| T285_RS01845 | rlmB | -0.41 | 0.001 |
| T285_RS08280 | - | -0.66 | 0.001 |
| T285_RS06755 | - | 0.42 | 0.001 |
| T285_RS06985 | nhaC | 0.96 | 0.001 |
| T285_RS02710 | - | 0.47 | 0.001 |
| T285_RS03140 | - | 0.56 | 0.001 |
| T285_RS05285 | - | 1.02 | 0.001 |
| T285_RS00520 | - | 0.67 | 0.001 |
| T285_RS09250 | - | 1.36 | 0.002 |
| T285_RS04460 | - | 0.91 | 0.002 |
| T285_RS01610 | infA | -0.34 | 0.002 |
| T285_RS03015 | - | -0.31 | 0.002 |
| T285_RS00670 | - | 0.67 | 0.002 |
| T285_RS01340 | - | 0.96 | 0.002 |
| T285_RS04115 | - | 0.41 | 0.002 |
| T285_RS01655 | rplM | -0.54 | 0.002 |
| T285_RS08440 | - | -2.05 | 0.002 |
| T285_RS04770 | - | 1.11 | 0.002 |
| T285_RS00890 | rpsN | -1.21 | 0.002 |
| T285_RS06715 | - | -0.76 | 0.002 |
| T285_RS05020 | - | 0.69 | 0.002 |
| T285_RS02300 | murI | -0.31 | 0.002 |
| T285_RS04605 | nth | 1.06 | 0.002 |
| T285_RS02980 | - | 0.44 | 0.002 |
| T285_RS08055 | - | -1.17 | 0.002 |
| T285_RS08850 | rpmH | -1.65 | 0.002 |
| T285_RS01335 | - | 1.88 | 0.002 |
| T285_RS00010 | dnaN | -0.42 | 0.002 |
| T285_RS07795 | - | -0.54 | 0.002 |
| T285_RS07055 | - | -0.50 | 0.002 |
| T285_RS05655 | - | 2.23 | 0.002 |
| T285_RS03005 | rbsK | 0.40 | 0.002 |
| T285_RS01325 | - | 0.85 | 0.002 |
| T285_RS07230 | - | -0.57 | 0.002 |
| T285_RS04245 | - | 1.03 | 0.002 |
| T285_RS08120 | - | -0.48 | 0.002 |
| T285_RS01310 | - | -0.43 | 0.002 |
| T285_RS05745 | - | 0.43 | 0.002 |
| T285_RS01765 | asp3 | -0.48 | 0.002 |
| T285_RS07405 | - | -2.11 | 0.002 |
| T285_RS06305 | galE | 0.45 | 0.002 |
| T285_RS06030 | - | -0.58 | 0.002 |
| T285_RS03120 | - | -0.53 | 0.003 |
| T285_RS08110 | ychF | -0.26 | 0.003 |
| T285_RS01815 | - | 0.54 | 0.003 |
| T285_RS04010 | - | 0.92 | 0.003 |
| T285_RS06375 | recA | -0.44 | 0.003 |
| T285_RS03430 | - | 0.65 | 0.003 |
| T285_RS03825 | recJ | 0.66 | 0.003 |
| T285_RS07140 | - | -0.43 | 0.003 |
| T285_RS00740 | - | -0.78 | 0.003 |
| T285_RS01840 | - | -0.97 | 0.003 |
| T285_RS06750 | - | 2.17 | 0.003 |
| T285_RS04260 | pyrR | 1.06 | 0.003 |
| T285_RS02200 | - | 1.27 | 0.003 |
| T285_RS04205 | - | 0.95 | 0.003 |
| T285_RS08210 | - | -0.55 | 0.003 |
| T285_RS05720 | mreD | 2.22 | 0.003 |
| T285_RS03085 | - | 0.32 | 0.003 |
| T285_RS01505 | rplD | -0.36 | 0.003 |
| T285_RS08905 | - | 1.71 | 0.003 |
| T285_RS07985 | - | -0.31 | 0.003 |
| T285_RS02325 | - | 0.80 | 0.003 |
| T285_RS03895 | - | 0.58 | 0.004 |
| T285_RS08270 | - | -0.62 | 0.004 |
| T285_RS01440 | dusB | -0.63 | 0.004 |
| T285_RS02970 | pstA | 0.99 | 0.004 |
| T285_RS07410 | - | -2.48 | 0.004 |
| T285_RS05025 | - | -0.54 | 0.004 |
| T285_RS07730 | mscL | -0.64 | 0.004 |
| T285_RS04500 | - | 0.94 | 0.004 |
| T285_RS05785 | rpsD | -0.69 | 0.004 |
| T285_RS05610 | - | -0.84 | 0.004 |
| T285_RS02875 | - | 1.47 | 0.004 |
| T285_RS00620 | - | -0.48 | 0.004 |
| T285_RS03595 | - | 0.39 | 0.004 |
| T285_RS00595 | - | -0.35 | 0.004 |
| T285_RS03735 | - | 0.41 | 0.004 |
| T285_RS07350 | adhE | 0.34 | 0.004 |
| T285_RS05765 | - | -0.64 | 0.004 |
| T285_RS03605 | ffh | 0.43 | 0.004 |
| T285_RS08900 | - | 0.51 | 0.004 |
| T285_RS04285 | pyrR | 1.25 | 0.004 |
| T285_RS00990 | - | -0.44 | 0.004 |
| T285_RS00390 | - | -0.45 | 0.004 |
| T285_RS01160 | - | 1.40 | 0.004 |
| T285_RS04035 | msrA | 0.79 | 0.005 |
| T285_RS08770 | - | -0.40 | 0.005 |
| T285_RS04180 | - | 0.83 | 0.005 |
| T285_RS03800 | grpE | 0.41 | 0.005 |
| T285_RS02350 | pepV | 0.50 | 0.005 |
| T285_RS05710 | mraZ | 0.62 | 0.005 |
| T285_RS02975 | pstB | 0.44 | 0.005 |
| T285_RS07115 | - | -0.38 | 0.005 |
| T285_RS04850 | - | 0.89 | 0.005 |
| T285_RS07200 | - | -0.45 | 0.005 |
| T285_RS00045 | - | -0.87 | 0.005 |
| T285_RS01490 | fusA | 0.36 | 0.005 |
| T285_RS00775 | - | -0.60 | 0.005 |
| T285_RS00375 | - | -0.81 | 0.005 |
| T285_RS05605 | mnmA | -0.46 | 0.005 |
| T285_RS00685 | phnE | 0.56 | 0.005 |
| T285_RS01495 | rpsJ | -1.52 | 0.005 |
| T285_RS04120 | - | 0.57 | 0.005 |
| T285_RS03935 | - | 0.92 | 0.005 |
| T285_RS04400 | - | 1.23 | 0.006 |
| T285_RS05210 | - | 0.88 | 0.006 |
| T285_RS04740 | recQ | 0.95 | 0.006 |
| T285_RS05935 | - | 0.45 | 0.006 |
| T285_RS03280 | - | 0.32 | 0.006 |
| T285_RS05905 | - | -0.36 | 0.006 |
| T285_RS03370 | - | 1.81 | 0.006 |
| T285_RS07750 | - | -0.33 | 0.006 |
| T285_RS04385 | - | 1.92 | 0.006 |
| T285_RS05995 | - | -0.44 | 0.006 |
| T285_RS03090 | - | 0.58 | 0.006 |
| T285_RS06785 | - | -0.26 | 0.006 |
| T285_RS04450 | - | 1.21 | 0.006 |
| T285_RS00910 | - | -0.82 | 0.007 |
| T285_RS06445 | - | -0.75 | 0.007 |
| T285_RS00405 | htpX | 0.40 | 0.007 |
| T285_RS04730 | - | 0.83 | 0.007 |
| T285_RS04890 | - | -0.91 | 0.007 |
| T285_RS00485 | - | -0.38 | 0.007 |
| T285_RS03235 | - | 0.42 | 0.007 |
| T285_RS03645 | lexA | -0.39 | 0.007 |
| T285_RS06965 | - | -0.67 | 0.007 |
| T285_RS05565 | typA | -0.32 | 0.007 |
| T285_RS01510 | rplW | -0.55 | 0.008 |
| T285_RS08125 | - | -0.60 | 0.008 |
| T285_RS05690 | - | 0.90 | 0.008 |
| T285_RS05430 | - | 0.23 | 0.008 |
| T285_RS03515 | - | 0.82 | 0.008 |
| T285_RS02850 | - | -0.39 | 0.008 |
| T285_RS08995 | - | 1.46 | 0.008 |
| T285_RS08525 | - | -0.46 | 0.008 |
| T285_RS06145 | - | 0.37 | 0.008 |
| T285_RS00435 | - | -0.61 | 0.008 |
| T285_RS08130 | noc | -0.48 | 0.009 |
| T285_RS08295 | - | -1.17 | 0.009 |
| T285_RS08805 | - | 0.45 | 0.009 |
| T285_RS06915 | - | 0.41 | 0.009 |
| T285_RS09300 | - | 1.42 | 0.009 |
| T285_RS05420 | - | 0.32 | 0.009 |
| T285_RS06625 | - | 0.72 | 0.009 |
| T285_RS04805 | - | 0.41 | 0.009 |
| T285_RS07025 | - | -0.93 | 0.009 |
| T285_RS05350 | rfbB | -0.80 | 0.009 |
| T285_RS04225 | - | 1.01 | 0.009 |
| T285_RS03840 | cls | 0.44 | 0.01 |
| T285_RS05345 | rfbA | -0.51 | 0.01 |
| T285_RS07075 | - | -0.78 | 0.01 |
| T285_RS02995 | - | 0.72 | 0.01 |
| T285_RS06745 | - | 0.94 | 0.01 |
| T285_RS07150 | tagD | -0.47 | 0.01 |
| T285_RS04485 | - | 0.94 | 0.01 |
| T285_RS03620 | trmD | 0.24 | 0.01 |
| T285_RS03690 | - | 0.46 | 0.01 |
| T285_RS07220 | - | -0.37 | 0.01 |
| T285_RS03175 | - | 0.52 | 0.01 |
| T285_RS04410 | - | 1.26 | 0.01 |
| T285_RS05090 | DegV2 | 0.31 | 0.01 |
| T285_RS05175 | - | -0.81 | 0.01 |
| T285_RS02940 | - | 0.39 | 0.01 |
| T285_RS03845 | - | 0.57 | 0.01 |
| T285_RS03945 | - | 0.50 | 0.01 |
| T285_RS06830 | - | 0.51 | 0.01 |
| T285_RS04160 | - | 0.97 | 0.01 |
| T285_RS00425 | - | 0.94 | 0.01 |
| T285_RS04920 | - | -0.63 | 0.01 |
| T285_RS00765 | - | -0.41 | 0.01 |
| T285_RS03315 | - | 3.57 | 0.01 |
| T285_RS00420 | - | 1.05 | 0.01 |
| T285_RS06735 | infC | -0.50 | 0.01 |
| T285_RS05820 | yidD | -0.42 | 0.01 |
| T285_RS05830 | - | -0.71 | 0.01 |
| T285_RS08645 | cydC | -0.57 | 0.01 |
| T285_RS07185 | - | -0.67 | 0.01 |
| T285_RS02635 | - | 0.58 | 0.01 |
| T285_RS02855 | - | -0.45 | 0.01 |
| T285_RS04390 | - | 0.88 | 0.01 |
| T285_RS03865 | - | 0.43 | 0.01 |
| T285_RS01030 | - | -0.77 | 0.01 |
| T285_RS07895 | - | -0.26 | 0.01 |
| T285_RS06245 | - | 0.81 | 0.01 |
| T285_RS02095 | - | 1.52 | 0.01 |
| T285_RS02035 | - | 0.53 | 0.01 |
| T285_RS06740 | - | 0.93 | 0.01 |
| T285_RS01790 | - | -0.93 | 0.01 |
| T285_RS06160 | - | 0.39 | 0.01 |
| T285_RS08600 | - | -0.53 | 0.01 |
| T285_RS08625 | - | -0.22 | 0.01 |
| T285_RS06450 | - | -0.58 | 0.01 |
| T285_RS05060 | topA | -0.22 | 0.02 |
| T285_RS08410 | - | 0.97 | 0.02 |
| T285_RS06970 | rlmD | 0.28 | 0.02 |
| T285_RS00175 | - | 0.94 | 0.02 |
| T285_RS02165 | - | -0.69 | 0.02 |
| T285_RS00950 | - | -0.33 | 0.02 |
| T285_RS07685 | - | 1.21 | 0.02 |
| T285_RS04130 | - | 0.43 | 0.02 |
| T285_RS00730 | nrdD | -0.42 | 0.02 |
| T285_RS01390 | - | -0.74 | 0.02 |
| T285_RS07935 | - | -0.86 | 0.02 |
| T285_RS01120 | - | -0.52 | 0.02 |
| T285_RS06330 | - | 0.53 | 0.02 |
| T285_RS08745 | - | -0.50 | 0.02 |
| T285_RS02495 | - | 0.60 | 0.02 |
| T285_RS02365 | - | -0.40 | 0.02 |
| T285_RS04510 | - | 0.67 | 0.02 |
| T285_RS05540 | - | 0.44 | 0.02 |
| T285_RS05415 | - | 0.40 | 0.02 |
| T285_RS01215 | - | -0.76 | 0.02 |
| T285_RS05760 | - | -0.61 | 0.02 |
| T285_RS00945 | - | -0.25 | 0.02 |
| T285_RS06645 | pheS | -0.42 | 0.02 |
| T285_RS04175 | lepB | 0.99 | 0.02 |
| T285_RS04905 | sufD | 0.31 | 0.02 |
| T285_RS01685 | - | -0.58 | 0.02 |
| T285_RS06470 | - | -0.56 | 0.02 |
| T285_RS01125 | - | -0.60 | 0.02 |
| T285_RS04840 | - | 0.93 | 0.02 |
| T285_RS05035 | - | 0.45 | 0.02 |
| T285_RS06955 | - | -0.46 | 0.02 |
| T285_RS07240 | - | -0.49 | 0.02 |
| T285_RS03360 | - | 0.68 | 0.02 |
| T285_RS06710 | yqeH | -0.40 | 0.02 |
| T285_RS04455 | - | 1.27 | 0.02 |
| T285_RS07275 | tnpA | -0.73 | 0.02 |
| T285_RS03485 | - | 0.63 | 0.02 |
| T285_RS02540 | - | -0.77 | 0.02 |
| T285_RS02280 | - | 1.18 | 0.02 |
| T285_RS04280 | carB | 0.62 | 0.02 |
| T285_RS07170 | - | -1.67 | 0.02 |
| T285_RS06655 | - | -0.27 | 0.02 |
| T285_RS02750 | - | 0.22 | 0.02 |
| T285_RS06880 | - | 0.36 | 0.02 |
| T285_RS07455 | - | -0.78 | 0.02 |
| T285_RS02745 | - | 0.42 | 0.02 |
| T285_RS03870 | - | 0.82 | 0.02 |
| T285_RS06355 | - | -0.62 | 0.02 |
| T285_RS05510 | - | 0.30 | 0.02 |
| T285_RS08340 | thiD | -0.74 | 0.02 |
| T285_RS03215 | - | 1.05 | 0.02 |
| T285_RS05740 | - | 1.18 | 0.02 |
| T285_RS03680 | pmtA | 0.30 | 0.02 |
| T285_RS04165 | - | 1.31 | 0.02 |
| T285_RS07505 | - | -1.97 | 0.02 |
| T285_RS03490 | rsmB | 0.46 | 0.02 |
| T285_RS01860 | - | -0.44 | 0.02 |
| T285_RS05195 | pyk | 0.31 | 0.02 |
| T285_RS07615 | - | 0.54 | 0.02 |
| T285_RS02270 | ruvX | 0.38 | 0.02 |
| T285_RS00920 | - | 0.24 | 0.02 |
| T285_RS08970 | - | -0.56 | 0.02 |
| T285_RS05940 | - | 1.65 | 0.02 |
| T285_RS03425 | - | 0.47 | 0.02 |
| T285_RS01015 | - | 0.77 | 0.02 |
| T285_RS00880 | - | -0.35 | 0.03 |
| T285_RS04790 | - | 0.90 | 0.03 |
| T285_RS07720 | - | -0.37 | 0.03 |
| T285_RS00645 | - | -0.68 | 0.03 |
| T285_RS03335 | - | 0.45 | 0.03 |
| T285_RS08500 | dltA | 0.24 | 0.03 |
| T285_RS03575 | - | 0.72 | 0.03 |
| T285_RS01850 | - | -0.53 | 0.03 |
| T285_RS00580 | - | -0.24 | 0.03 |
| T285_RS08315 | - | -0.39 | 0.03 |
| T285_RS02900 | - | -0.37 | 0.03 |
| T285_RS01755 | asp1 | -0.42 | 0.03 |
| T285_RS01500 | rplC | -0.88 | 0.03 |
| T285_RS01700 | - | -1.00 | 0.03 |
| T285_RS05900 | - | -0.41 | 0.03 |
| T285_RS02160 | - | -0.74 | 0.03 |
| T285_RS02700 | - | 0.75 | 0.03 |
| T285_RS01025 | - | -0.41 | 0.03 |
| T285_RS08250 | - | -0.87 | 0.03 |
| T285_RS02375 | - | -0.53 | 0.03 |
| T285_RS07700 | - | -0.83 | 0.03 |
| T285_RS03830 | - | 0.74 | 0.03 |
| T285_RS02025 | - | -0.81 | 0.03 |
| T285_RS01980 | - | -0.47 | 0.03 |
| T285_RS01870 | - | -0.55 | 0.03 |
| T285_RS08180 | - | 0.34 | 0.03 |
| T285_RS08320 | - | -0.29 | 0.03 |
| T285_RS06520 | - | 0.88 | 0.03 |
| T285_RS00125 | - | 0.89 | 0.03 |
| T285_RS01525 | rplV | -0.41 | 0.03 |
| T285_RS07930 | - | -0.27 | 0.03 |
| T285_RS03075 | - | 0.54 | 0.03 |
| T285_RS08495 | dltB | 0.45 | 0.03 |
| T285_RS05095 | - | 0.37 | 0.03 |
| T285_RS08030 | - | -0.78 | 0.03 |
| T285_RS02240 | - | -0.43 | 0.03 |
| T285_RS03555 | - | 0.44 | 0.03 |
| T285_RS08925 | - | 0.68 | 0.03 |
| T285_RS08790 | - | 0.46 | 0.03 |
| T285_RS00655 | - | -0.54 | 0.03 |
| T285_RS02760 | - | -0.41 | 0.03 |
| T285_RS02685 | - | 1.00 | 0.03 |
| T285_RS02955 | - | -0.78 | 0.03 |
| T285_RS09035 | - | 1.65 | 0.03 |
| T285_RS02065 | - | 0.55 | 0.03 |
| T285_RS00695 | - | -0.32 | 0.03 |
| T285_RS01595 | rplO | -0.23 | 0.03 |
| T285_RS03855 | - | 0.39 | 0.03 |
| T285_RS03910 | - | 0.92 | 0.04 |
| T285_RS05215 | - | 0.28 | 0.04 |
| T285_RS08455 | - | -0.39 | 0.04 |
| T285_RS06380 | pgsA | 0.32 | 0.04 |
| T285_RS01795 | - | -0.64 | 0.04 |
| T285_RS08325 | - | -0.88 | 0.04 |
| T285_RS01590 | rpmD | -0.26 | 0.04 |
| T285_RS00600 | - | -0.27 | 0.04 |
| T285_RS02395 | - | 0.69 | 0.04 |
| T285_RS00165 | - | -0.77 | 0.04 |
| T285_RS03065 | - | 0.27 | 0.04 |
| T285_RS07815 | - | -0.51 | 0.04 |
| T285_RS00430 | - | 0.61 | 0.04 |
| T285_RS03355 | - | 1.35 | 0.04 |
| T285_RS01745 | - | -0.46 | 0.04 |
| T285_RS01055 | trpS | -0.43 | 0.04 |
| T285_RS06780 | nrdR | -0.42 | 0.04 |
| T285_RS09245 | - | 0.83 | 0.04 |
| T285_RS02820 | - | 0.48 | 0.04 |
| T285_RS06435 | - | -0.52 | 0.04 |
| T285_RS03525 | - | 0.54 | 0.04 |
| T285_RS04835 | - | 0.76 | 0.04 |
| T285_RS01155 | - | 0.63 | 0.04 |
| T285_RS03255 | glmS | -0.31 | 0.04 |
| T285_RS01825 | radA | 0.52 | 0.04 |
| T285_RS03270 | - | 0.35 | 0.04 |
| T285_RS02435 | - | -0.21 | 0.04 |
| T285_RS07175 | - | -0.63 | 0.04 |
| T285_RS05590 | - | 0.39 | 0.04 |
| T285_RS02690 | - | 0.44 | 0.04 |
| T285_RS02835 | - | 0.71 | 0.04 |
| T285_RS05770 | thiI | -0.49 | 0.04 |
| T285_RS05470 | - | -0.46 | 0.04 |
| T285_RS05105 | - | 0.28 | 0.04 |
| T285_RS08415 | manX | -0.27 | 0.04 |
| T285_RS00935 | - | 0.44 | 0.04 |
| T285_RS00965 | greA | 0.54 | 0.04 |
| T285_RS03980 | - | 0.97 | 0.04 |
| T285_RS05260 | - | 0.71 | 0.04 |
| T285_RS07820 | - | -0.68 | 0.04 |
| T285_RS00760 | - | -0.64 | 0.04 |
| T285_RS08225 | - | 1.03 | 0.04 |
| T285_RS04025 | msrB | 0.72 | 0.05 |
| T285_RS06075 | - | 0.94 | 0.05 |
| T285_RS00130 | - | -0.49 | 0.05 |
| T285_RS01915 | - | -0.75 | 0.05 |
| T285_RS03965 | - | 0.58 | 0.05 |
| T285_RS06035 | - | 0.96 | 0.05 |
| T285_RS02460 | - | 0.23 | 0.05 |
| T285_RS07725 | - | 0.38 | 0.05 |
| T285_RS03240 | - | 0.98 | 0.05 |
